# Metal-free Minisci C–H alkylation of hydrazones using aldehydes: an unexpected route to hydrazone-containing pyrimidine derivatives

**DOI:** 10.1039/d6ra01465h

**Published:** 2026-04-29

**Authors:** Atefeh Tirehdast, Volodymyr Semeniuchenko, Ali Shiri

**Affiliations:** a Department of Chemistry, Faculty of Science, Ferdowsi University of Mashhad Mashhad Iran alishiri@um.ac.ir; b Department of Chemistry and Biomolecular Sciences, Faculty of Science, University of Ottawa Ottawa Canada

## Abstract

In this research, a novel method for synthesizing various heterocyclic derivatives *via* the Minisci radical substitution reaction has been presented. The Minisci reaction offers a very efficient and valuable approach for directly functionalizing heteroarenes with an electron-deficient group, allowing the formation of carbon–carbon bonds straightforwardly and efficiently. We have developed a metal-free, light-independent Minisci-type strategy for the direct C–H functionalization of pyrimidines, thereby facilitating the design of heteroaromatic derivatives containing hydrazone groups. The target derivatives were successfully synthesized in good yields using aromatic aldehydes as alkyl radical precursors. The reaction proceeds through a sequence of hydrogen atom abstraction (HAA) from the aldehyde, followed by decarbonylation under oxidative conditions using K_2_S_2_O_8_ and trifluoroacetic acid (TFA). This method provides a practical and scalable approach to accessing structurally heterocyclic compounds of various classes.

## Abstract 2

A practical method for metal-free, Minisci-type decarbonylative functionalization of pyrimidines using aldehydes has been developed, yielding hydrazone derivatives.

## Graphical abstract

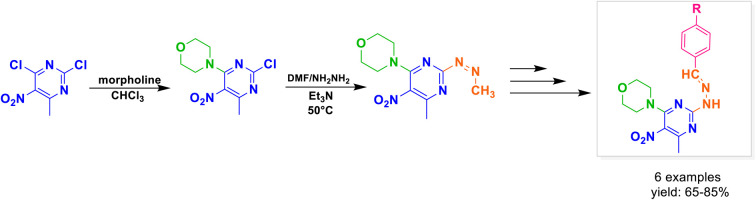

## Introduction

The Minisci reaction, first developed in the late 1960s by Francesco Minisci, is a significant strategy for the direct radical functionalization of electron-deficient nitrogen-containing heteroarenes.^1–5^ One of the key advantages of this process is its ability to directly attach alkyl and aryl groups onto heteroaromatic frameworks without needing prior substrate activation.^6–11^ This notable property has made the Minisci reaction essential for the synthesis of complex bioactive compounds and producing pharmaceuticals and important synthetic intermediates.^12–14^

Even if there are such merits, classical Minisci protocols generally experience inherent deficiencies. The traditional protocols generally rely on metal catalysis, photochemical initiation, or stoichiometric amounts of strong oxidants.^15^ Such needs not only become prohibitively expensive and environmentally unfavorable but also impose severe limitations on scalability and functional group toleration.^16–19^ Consequently, efforts in the recent past were aimed at the synthesis of more practical alternatives. Metal-free and photocatalyst-free forms became the future Minisci-type protocols through the accomplishment of the conversion under milder, more sustainable conditions, in alignment with the principles of the green chemistry movement.^20–24^ Of the many radical precursors studied for Minisci reactions, the aldehyde case is particularly unique.^25^ It simplifies the process by inclusion of aldehydes, and offers access to a vast new product range.^26–28^

Recent studies have demonstrated that aldehydes can serve as effective radical precursors in Minisci-type C–H functionalization of heteroarenes. In several reported systems, these transformations proceed under visible-light irradiation through photochemically initiated radical pathways.^29^ In addition, related approaches have utilized metal catalysts in combination with oxidants to enable oxidative C–H functionalization using aldehydes as coupling partners.^30^ While these studies highlight the versatility of aldehydes in radical C–H functionalization chemistry, many of the existing methods rely on either external light sources or metal catalysts. In contrast, the present study describes a metal-free and light-independent Minisci-type protocol for the C–H functionalization of pyrimidines using readily available aldehydes, providing straightforward access to hydrazone-containing heterocyclic derivatives.

**Fig. 1:**
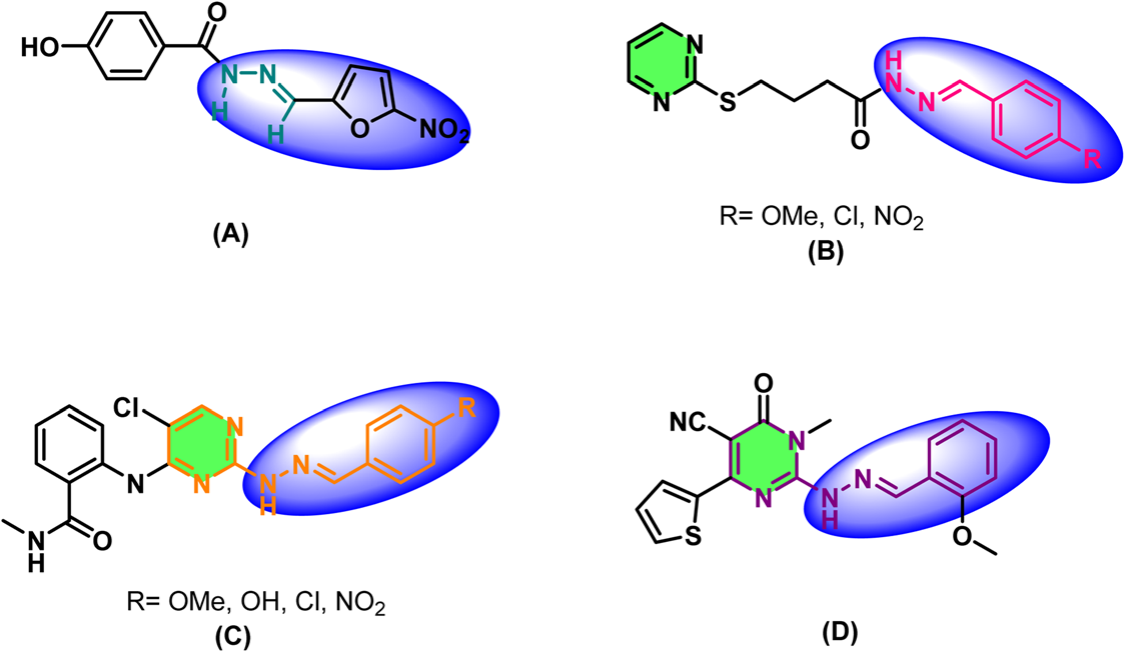
Representative natural products and drugs with hydrazone skeletons.

On the other hand, pyrimidines, among the nitrogen heterocycles, have long been a focus of medicinal chemists due to their vast array of biological activities, including anticancer,^31^ antiviral,^32,33^ antibacterial,^34,35^ and enzyme inhibitory properties.^36,37^ Their unique properties and strong ability to engage biological targets render them popular scaffolds in modern drug design. Moreover, hydrazone-containing frameworks have attracted significant attention owing to their diverse biological properties, including anticancer, anti-inflammatory, antifungal, and antiviral activities.^38,39^ The incorporation of the hydrazone substructures on heteroaromatic rings such as pyrimidines has the potential to endow heterocycles with promising pharmacological properties. One among the subsets of the hydrazone compounds occupies central roles in various drugs and natural products, for instance, nifuroxazide (A) functions as an intestinal antiseptic; compounds (B), exhibit anti-tubercular activity, compounds (C) show anti-thyroid cancer properties, while compound (D) displays analgesic and antibacterial activities (Fig. 1).^40–43^

Motivated by such remarks and in continuation of our previous studies,^44–47^ the present work looks at the potential use of aldehydes as radical precursor sources in the synthesis of hydrazone-containing heterocyclic derivatives *via* Minisci-type reactions. This work attempts to offer a convenient, metal-free, and light-independent synthetic process that not only adheres to the principles of green chemistry but also offers a viable process for accessing novel heteroaromatic hydrazones of remarkable pharmaceutical promise.

## Results and discussion

**Scheme 1:**
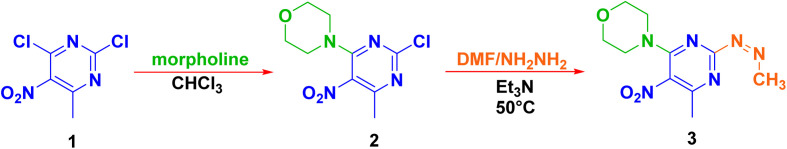
The schematic preparation of compound 3.

For the synthesis of heterocycles 4a–f, initially, 2,4-dichloro-6-methyl-5-nitropyrimidine (1, synthesized by known method)^48^ was stirred at room temperature with morpholine in chloroform for 2 h to obtain precursor 2.^49^ In the subsequent investigation, we hypothesized that the 2, when treated with hydrazine hydrate in a mixture of triethylamine (Et_3_N) and dimethylformamide (DMF), would undergo direct hydrazination. However, analysis revealed the formation of compound 3 (Scheme 1).

**Scheme 2:**
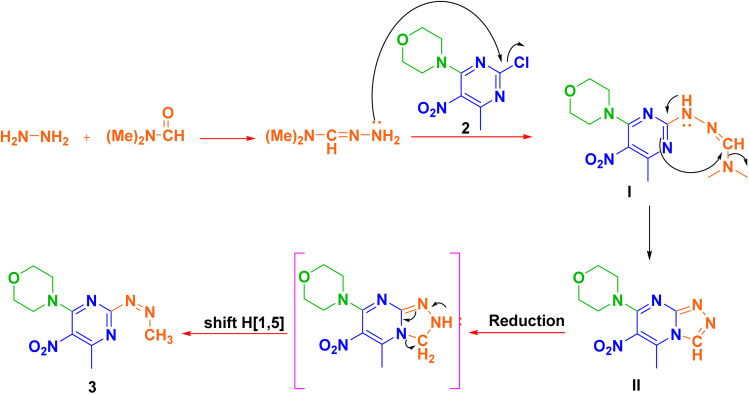
Plausible mechanism for the synthesis of compound 3.

Several intermediates were isolated at different time points and their structures confirmed through spectral analysis. In the proposed mechanism, hydrazine hydrate and DMF initially react, and the resulting product acts as a nucleophile attacking the chlorine atom in compound 2 at position 4, forming structure I.^50–52^ Then the reaction leads to the formation of a pyrazole ring, as in compound II. The presence of hydrazine hydrate subsequently causes the CH=N double bond to be reduced.^53–55^ Through sigmatropic shift of H[1,5] under thermal conditions a more stable product forms by rearranging π bonds, ultimately leading to the final compound 3 (Scheme 2).

In order to assay the Minisci reaction, the optimization studies were carried out using compound 3 and 4-isopropylbenzaldehyde as model substrates (Table 1). We investigated different oxidants and solvents to find suitable reaction conditions. Experiments conducted in acetonitrile, whether using trifluoroacetic acid (TFA) or sulfuric acid (H_2_SO_4_) (entries 1 and 2), using ammonium persulfate as the oxidant and dichloroethane (DCE) as the solvent, resulted in negligible product formation (entry 3), while replacing TFA with H_2_SO_4_ under the same conditions (entry 4) completely inhibited the reaction. In contrast, the combination of DCE, TFA, and potassium persulfate produced the desired product (yield = 85%) (entry 5), indicating that these conditions were optimal. The superior performance of K_2_S_2_O_8_ compared to (NH_4_)_2_S_2_O_8_ may be attributed to its higher stability under the reaction conditions, which likely enables a more efficient and controlled generation of sulfate radical species. Alternative oxidants, such as benzoyl peroxide (entry 6), and solvents, such as DMF (entry 7), failed to improve the reaction efficiency. Furthermore, reactions conducted in DCE without the addition of acid (entry 8) or in the absence of solvent (entry 9) did not yield the desired product. The reaction was also performed at room temperature (entry 10); however, no product was observed, likely due to insufficient thermal activation of K_2_S_2_O_8_ required to generate sulfate radical species. Overall, these studies identify dichloroethane as the appropriate solvent, trifluoroacetic acid as the acid, and potassium persulfate as the optimal oxidant in reflux conditions.

**Table 1:** The optimization of the reaction conditions

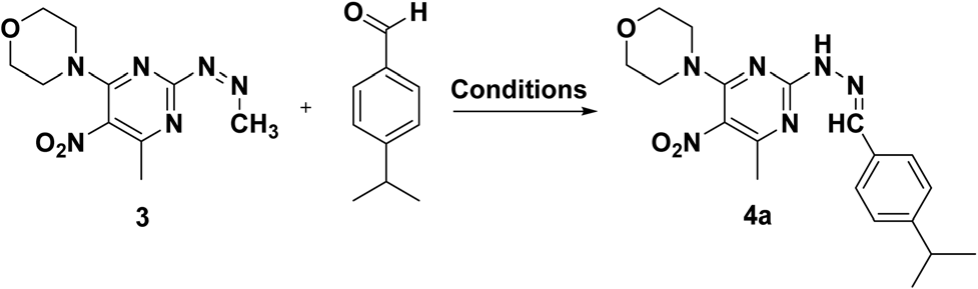						
Entry	Solvent	Acid	Oxidant	Time (h)	Temperature (°C)	Yield (%)
1	CH_3_CN	TFA	K_2_S_2_O_8_	24	Reflux	—
2	CH_3_CN	H_2_SO_4_	K_2_S_2_O_8_	24	Reflux	—
3	DCE	TFA	(NH_4_)_2_S_2_O_8_	20	Reflux	Trace
4	DCE	H_2_SO_4_	(NH_4_)_2_S_2_O_8_	24	Reflux	—
**5**	**DCE**	**TFA**	**K**_**2**_ **S**_**2**_ **O**_**8**_	**16**	**Reflux**	**85**
6	DCE	TFA	Benzoyl peroxide	24	Reflux	—
7	DMF	TFA	K_2_S_2_O_8_	24	100	—
8	DCE	—	K_2_S_2_O_8_	24	Reflux	—
9	—	TFA	K_2_S_2_O_8_	24	100	—
10	DCE	TFA	K_2_S_2_O_8_	24	25	—

**Scheme 3:**
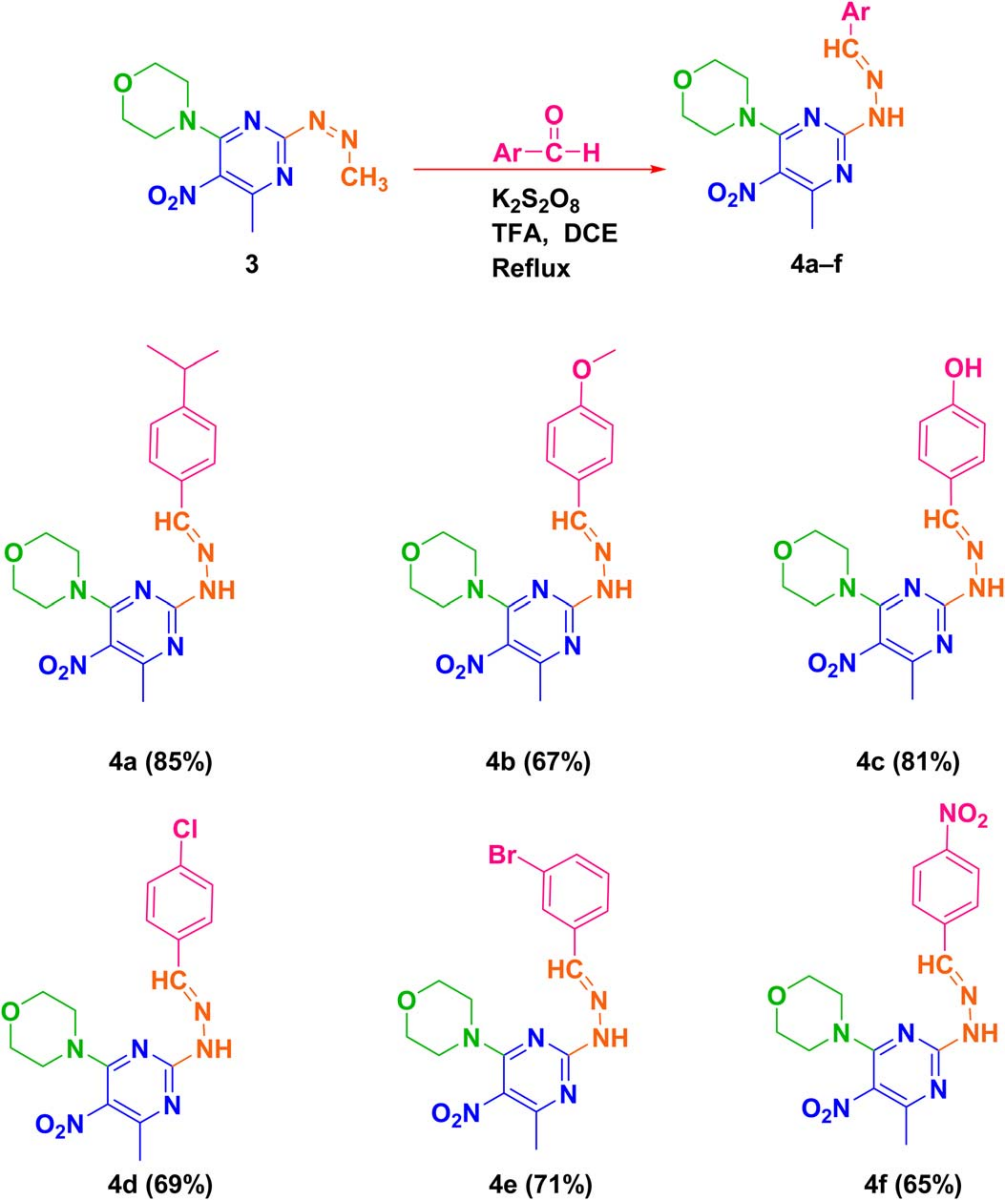
The schematic preparation of derivatives 4a–f.

In the final step, products 4a–f were synthesized by reacting compound 3 with various aldehyde derivatives in K_2_S_2_O_8_ as a radical source, TFA as an acid catalyst, and dichloroethane as solvent under reflux conditions (Scheme 3).

**Fig. 2:**
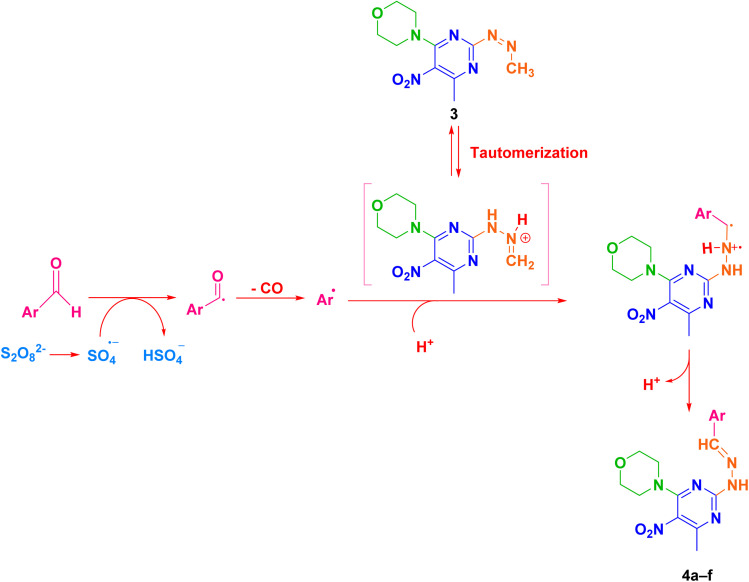
The plausible mechanism for the synthesis of compounds 4a–f.

Based on the literature reports^56,57^ and our studies, a plausible mechanism is proposed in Fig. 2. The Minisci reaction involves radical C–H functionalization of electron-deficient heteroarenes, in which aldehydes act as precursors to alkyl radicals *via* a decarbonylation pathway. Protonation of compound 3 with trifluoroacetic acid (TFA) increases its electrophilicity and directs radical addition to the most electron-deficient sites. Initially, potassium persulfate decomposes under the reaction conditions to generate sulfate radicals, which initiate the transformation of aldehydes into alkyl radicals through a sequence of radical steps. Mechanistically, the sulfate radical first abstracts the formyl hydrogen of the aldehyde, yielding a transient acyl radical intermediate. This intermediate rapidly undergoes decarbonylation, extruding CO to furnish the corresponding alkyl radical. The alkyl radical then adds to the protonated heteroarene to form a radical cation, which ultimately provides the products 4a–f.

**Fig. 3:**
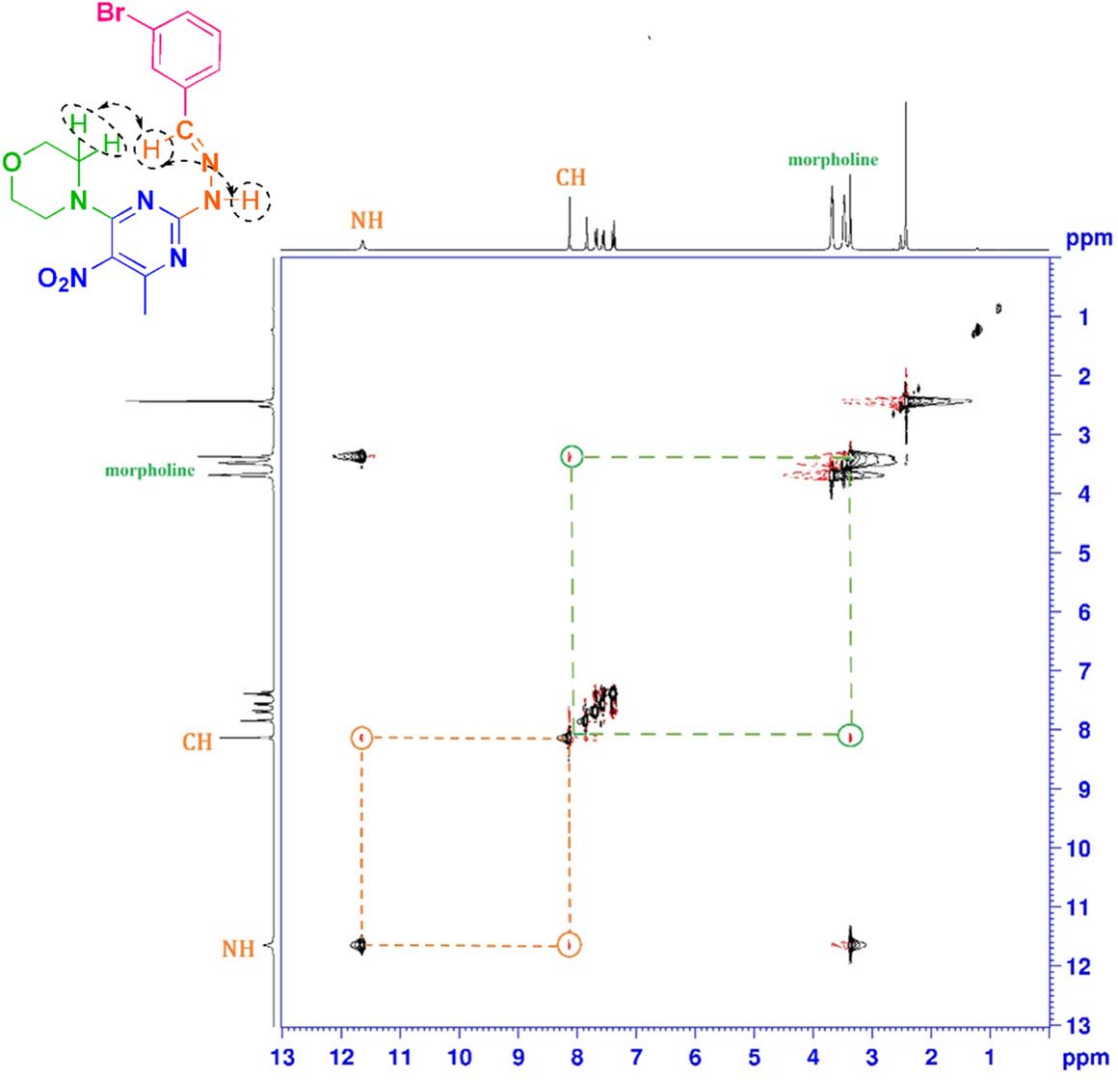
Two-dimensional NOESY spectrum of compound 4e.

All the structures were characterized, and the spectral data of one of the synthesized derivatives, 4-(2-(2-(4-isopropylbenzylidene)hydrazineyl)-6-methyl-5-nitropyrimidin-4-yl)morpholine 4a, were reported. In the IR spectrum of this derivative, the stretching vibration bands appear at *ν*_max_ 3277 cm^−1^ (NH), whereas the IR spectrum of compound 3 reveals the absence of an NH moiety. The C–H stretching vibrations of both aromatic and aliphatic groups appear in the range of *ν* = 2868–3133 cm^−1^, and symmetric and asymmetric stretching vibrations of the nitro group in the range of *ν* = 1573, 1327 cm^−1^. In the ^1^H NMR spectrum, a doublet signal at *δ* 1.23 ppm corresponds to six hydrogens of the isopropyl moiety (–CH(CH̲_3_)_2_). There is a single signal at *δ* 2.44 ppm corresponding to three hydrogens of the methyl of the pyrimidine ring, and a peak as a multiplet integrating for one hydrogen is associated, corresponding to (–CH̲(CH_3_)_2_) and two peaks appear as triplets in *δ* 3.48 ppm and *δ* 3.69 ppm, each integrating for four hydrogens, confirm the presence of the morpholine ring. Two peaks as a doublet signal at *δ* 7.32 ppm and *δ* 7.61 ppm, each integrating for two hydrogens, corresponded to the phenyl protons, and a signal at *δ* 8.16 ppm, corresponding to one hydrogen, is associated with (–CH=N). Also, the spectrum shows a singlet D_2_O-exchangeable signal at *δ* 11.53 ppm, indicating the NH moiety. In the ^13^C NMR spectrum, two groups of peaks are observed: five peaks in the aliphatic region corresponding to C̲H_3_-pyrimidine, and carbons of isopropyl and morpholine moieties. Nine signals appear in the aromatic region, eight of which correspond to the carbons of the pyrimidine and phenyl rings. In contrast, one signal at *δ* 144.4 ppm corresponds to the carbon of CH=N, confirming the true structure of this compound. Furthermore, Nuclear Overhauser Effect Spectroscopy (NOESY) was conducted on compound 4e. The two-dimensional NOESY spectrum, resembling the ^1^H NMR spectrum, confirmed the spatial relationship between the CH̲=N and NH protons, as evidenced by the presence of cross-peaks. This indicates that these two hydrogen atoms are in close proximity within the molecular structure. Additionally, the spatial relationship between the CH̲=N and the morpholine protons is confirmed by the presence of cross-peaks (Fig. 3).

Eventually, the mass spectrum of this sample shows the molecular ion peak at *m*/*z* = 384 and a peak at *m*/*z* = 219, which corresponds to the cleavage of the nitro and isopropylbenzene groups from the main structure, confirming the synthesis of the desired compound 4a. Attempted crystallography for compound 4a confirmed the connectivity of the atoms, but significant twinning did not allow the collection of publishable data for the crystal structure of this compound.

## Conclusion

In summary, a protocol for the preparation of new derivatives of pyrimidine containing hydrazone substituents 4a–f has been reported and obtained by the metal-free and light-independent Minisci reaction. The synthetic method used radical precursors in the form of aldehydes and an oxidative medium mediated by K_2_S_2_O_8_–TFA, providing an efficient method for the direct functionalization of the pyrimidine derivatives containing the hydrazone 4a–f*via* C–C cross-coupling. The derivatives obtained were carefully characterized by using spectroscopic and microanalytical methods to confirm the structural framework. Pyrimidine–hydrazone moieties are of great interest because they have a wide biological spectrum.

## Experimental

Melting points were measured by an Electrothermal Type 9200 melting point apparatus. The ^1^H NMR (300 MHz) and the ^13^C NMR (75 MHz) spectra were obtained on a Bruker Avance DRX-300 Fourier transfor spectrometer. An Avatar 370 FT-IR Thermo Nicolet spectrometer was employed to record the IR spectra, and a Varian Mat CH-7 instrument for collecting mass spectra with 70 eV. Microanalytical data were obtained on a Thermo Finnigan Flash EA 1112 microanalyzer.

### 2-Chloro-6-methyl-4-morpholino-5-nitropyrimidine (2)

Yellow needle crystals; yield = 90%; m. p. 115–116 °C; IR (KBr disc, cm^−1^): *ν* 3427, 2986, 2925, 2864, 1580, 1495, 1423, 1344, 1290, 1228, 1141. ^1^H NMR (300 MHz, CDCl_3_): *δ*_H_ 2.50 (s, 3H, CH_3_), 3.60 (t, *J* = 4.8 Hz, 4H, morpholine), 3.78–3.82 (m, 4H, morpholine). ^13^C NMR (75 MHz, CDCl_3_): *δ*_C_ 21.3, 46.6, 66.3, 131.0, 154.8, 158.8, 163.5. MS (*m*/*z*) = 258 (M^+^), 198 [M^+^ − (Me, NO_2_)], 128 [M^+^ − (morpholine, NO_2_)]. Anal. calcd for C_9_H_11_ClN_4_O_3_ (%): C, 41.79; H, 4.29; N, 21.66. Found: C, 41.77; H, 4.27; N, 21.64.

### General procedure for the synthesis of compound (3)

To prepare 6-methyl-2-(methyldiazenyl)-4-morpholino-5-nitropyrimidine 3, a solution of compound 2 (1 mmol, 0.258 g) in DMF (1 mL) was added to a mixture of hydrazine hydrate (0.24 mL, 5 mmol), triethylamine (0.3 mL, 2 mmol), and DMF (2 mL), which had been preheated to 50 °C. The resulting mixture was then stirred at 50 °C for 10 h. The progress of the reaction was monitored by TLC using chloroform : methanol (20 : 1) as eluent. After the completion of the reaction, the solvent was removed under reduced pressure, and the resulting solid was washed with water (2 × 20 mL) and dried.

### *N*,*N*-Dimethyl-*N*′-(4-methyl-6-morpholino-5-nitropyrimidin-2-yl)formohydrazonamide (I)

Brown powder; yield = 54%; m. p. 130–131 °C; IR (KBr disc, cm^−1^): *ν* 3338, 3260, 3203, 3039, 2960, 2900, 1634, 1567, 1511, 1470, 1442, 1330, 1263. ^1^H NMR (300 MHz, DMSO-*d*_6_): *δ*_H_ 2.26 (s, 3H, CH_3_-pyrimidine), 2.32–2.38 (m, 6H, N(CH_3_)_2_), 3.62–3.65 (m, 8H, morpholine), 5.80 (s, 1H, CH), 8.20 (s, 1H, NH). ^13^C NMR (75 MHz, DMSO-*d*_6_): *δ*_C_ 24.9, 37.2, 47.2, 66.2, 123.9, 143.3, 158.6, 158.7, 163.8. MS (*m*/*z*) = 309 (M^+^), 177 [M^+^ − (morpholine, NO_2_)]. Anal. calcd for C_12_H_19_N_7_O_3_ (%): C, 46.59; H, 6.19; N, 31.70. Found: C, 46.57; H, 6.17; N, 31.68.

### 4-(5-Methyl-6-nitro-[1,2,4]triazolo[4,3-*a*]pyrimidin-7-yl)morpholine (II)

Yellow powder; yield = 44%; m. p. 123–124 °C; IR (KBr disc, cm^−1^): *ν* 3208, 3139, 3090, 2988, 2898, 2864, 1585, 1524, 1480, 1420, 1379, 1358, 1307, 1280, 1187. MS (*m*/*z*) = 264 (M^+^), 218 [M^+^ − NO_2_], 178 [M^+^ − (morpholine)]. Anal. calcd for C_10_H_12_N_6_O_3_ (%): C, 45.45; H, 4.58; N, 31.80. Found: C, 45.43; H, 4.56; N, 31.78.

### 6-Methyl-2-(methyldiazenyl)-4-morpholino-5-nitropyrimidine (3)

Yellow powder; yield = 65%; m. p. 102–103 °C; IR (KBr disc, cm^−1^): *ν* 3035, 2970, 2925, 2858, 1558, 1486, 1445, 1402, 1370, 1323, 1267, 1232, 1141. ^1^H NMR (300 MHz, CDCl_3_): *δ*_H_ 2.51 (s, 3H, CH_3_-pyrimidine), 3.22 (s, 3H, CH_3_), 3.47–3.50 (m, 4H, morpholine), 3.76–3.79 (m, 4H, morpholine). ^13^C NMR (75 MHz, CDCl_3_): *δ*_C_ 23.6, 37.2, 47.2, 66.8, 124.3, 157.1, 158.7, 164.1. MS (*m*/*z*) = 266 (M^+^), 220 [M^+^ − NO_2_], 135 [M^+^ − (morpholine, NO_2_)]. Anal. calcd for C_10_H_14_N_6_O_3_ (%): C, 45.11; H, 5.30; N, 31.56. Found: C, 45.09; H, 5.28; N, 31.54.

### General procedure for the synthesis of compound (4a–f)

A mixture of 6-methyl-2-(methyldiazenyl)-4-morpholino-5-nitropyrimidine 3 (1 mmol, 0.266 g), various aldehyde derivatives (3 mmol), K_2_S_2_O_8_ (2.3 mmol, 0.621 g), and TFA (1 mmol, 0.07 mL) in DCE (3 mL) was heated under reflux for 16 h. After completion of the reaction, which was monitored by TLC using CHCl_3_ : MeOH (30 : 1), the solvent was evaporated under reduced pressure. Water (5 mL) was added, and the mixture was neutralized with an aqueous 5% NaHCO_3_ solution. The mixture was extracted with EtOAc (3 × 10 mL), and the organic phase was dried over anhydrous Na_2_SO_4_ and evaporated under reduced pressure. The crude product was purified by column chromatography on silica gel using CHCl_3_ : MeOH (30 : 1) as eluent to yield the pure product.

#### 4-(2-(2-(4-Isopropylbenzylidene)hydrazineyl)-6-methyl-5-nitropyrimidin-4-yl)morpholine (4a)

Yellow powder; yield = 85%; m. p. 168–169 °C; IR (KBr disc, cm^−1^): *ν* 3277, 3133, 2958, 2925, 2868, 1617, 1573, 1508, 1429, 1377, 1327, 1255. ^1^H NMR (300 MHz, DMSO-*d*_6_): *δ*_H_ 1.23 (d, *J* = 6.9 Hz, 6H, 2CH_3_), 2.44 (s, 3H, CH_3_-pyrimidine), 2.92 (m, 1H, CH), 3.48 (t, *J* = 4.8 Hz, 4H, morpholine), 3.69 (t, *J* = 4.7 Hz, 4H, morpholine), 7.32 (d, *J* = 8.1 Hz, 2Ar-H), 7.61 (d, *J* = 8.2 Hz, 2Ar-H), 8.16 (s, 1H, CH), 11.53 (s, 1H, NH, D_2_O exchangeable). ^13^C NMR (75 MHz, DMSO-*d*_6_): *δ*_C_ 23.2, 24.2, 33.8, 47.0, 66.2, 125.2, 127.2, 127.3, 132.9, 137.3, 144.4, 150.5, 157.1, 164.1. MS (*m*/*z*) = 384 (M^+^), 219 [M^+^ − (isopropylbenzene, NO_2_)]. Anal. calcd for C_19_H_24_N_6_O_3_ (%): C, 59.36; H, 6.29; N, 21.86. Found: C, 59.34; H, 6.27; N, 21.84.

Single crystals of compound 4a were grown by slow evaporation of acetonitrile solution. For all observed crystals non-merohedral twinning with the presence of multiple domains was observed, and a non-twinned crystal could not be found. Application of other solvents did not allow single crystal growth. X-ray diffraction data collection (Mo Kα radiation, 0.71073 Å wavelength) at 100 K temperature allowed to determine unit cell and space group (*a* = 11.9529 Å, *b* = 13.3518 Å, *c* = 15.7124 Å, *α* = 93.8315°, *β* = 111.2006°, *γ* = 113.9578°, space group *P*1̄). The asymmetric unit contained two molecules of 4a and one MeCN molecule. Although structure solution and refinement confirmed the atoms connectivity for compound 4a, final *R*-factors (*R*_1_ = 0.2704, w*R*_2_ = 0.4368) made the structure not publishable. High *R*-factors are observed due to crystal twinning and inability to detwin the data using TWINABS software (in Bruker APEX II suite). To our surprise, the structure has not shown any disorder, and all hydrogen atoms were resolved in the difference Fourier map after location and anisotropic refinement of heavy atoms. Located H atoms as well confirmed the structure 4a.

#### 4-(2-(2-(4-Methoxybenzylidene)hydrazineyl)-6-methyl-5-nitropyrimidin-4-yl)morpholine (4b)

Yellow powder; yield = 67%; m. p. 161–162 °C; IR (KBr disc, cm^−1^): *ν* 3284, 2966, 2904, 2843, 1609, 1571, 1534, 1510, 1433, 1374, 1329, 1250, 1166. ^1^H NMR (300 MHz, DMSO-*d*_6_): *δ*_H_ 2.43 (s, 3H, CH_3_-pyrimidine), 3.46–3.49 (m, 4H, morpholine), 3.67–3.70 (m, 4H, morpholine), 3.81 (m, 3H, O–CH_3_), 6.99–7.02 (m, 2H, 2Ar-H), 7.62–7.65 (m, 2H, 2Ar-H), 8.14 (s, 1H, CH), 11.46 (s, 1H, NH). ^13^C NMR (75 MHz, DMSO-*d*_6_): *δ*_C_ 23.3, 47.0, 55.7, 66.2, 108.8, 114.8, 125.4, 127.8, 128.7, 144.3, 157.1, 160.9, 164.2. MS (*m*/*z*) = 372 (M^+^), 220 [M^+^ − (methoxybenzene, NO_2_)], 133 [M^+^ − (methoxybenzene, morpholine, NO_2_)]. Anal. calcd for C_17_H_20_N_6_O_4_ (%): C, 54.83; H, 5.41; N, 22.57. Found: C, 54.81; H, 5.39; N, 22.55.

#### 4-((2-(4-Methyl-6-morpholino-5-nitropyrimidin-2-yl)hydrazineylidene)methyl)phenol (4c)

Yellow powder; yield = 81%; m. p. 214–215 °C; IR (KBr disc, cm^−1^): *ν* 3235, 2962, 2927, 2851, 1621, 1578, 1552, 1470, 1421, 1326, 1268, 1196. ^1^H NMR (300 MHz, DMSO-*d*_6_): *δ*_H_ 2.53 (s, 3H, CH_3_-pyrimidine), 3.68–3.71 (m, 8H, morpholine), 6.89–6.95 (m, 3H, (2Ar-H, OH)), 7.28 (t, *J* = 7.8 Hz, 1H, Ar-H), 7.43 (d, *J* = 7.5 Hz, 1H, Ar-H), 8.35 (s, 1H, CH), 11.88 (s, 1H, NH). ^13^C NMR (75 MHz, DMSO-*d*_6_): *δ*_C_ 23.2, 47.1, 66.1, 116.9, 119.2, 119.7, 125.8, 130.3, 131.1, 145.7, 156.6, 156.8, 157.9, 164.3. MS (*m*/*z*) = 358 (M^+^), 327 [M^+^ − (OH, Me)], 220 [M^+^ − (hydroxybenzol, NO_2_)]. Anal. calcd for C_16_H_18_N_6_O_4_ (%): C, 53.63; H, 5.06; N, 23.45. Found: C, 53.61; H, 5.04; N, 23.43.

#### 4-(2-(2-(4-Chlorobenzylidene)hydrazineyl)-6-methyl-5-nitropyrimidin-4-yl)morpholine (4d)

Yellow powder; yield = 69%; m. p. 224–225 °C; IR (KBr disc, cm^−1^): *ν* 3108, 2963, 2894, 2857, 1598, 1583, 1528, 1486, 1431, 1335, 1257, 1164. ^1^H NMR (300 MHz, DMSO-*d*_6_): *δ*_H_ 2.44 (s, 3H, CH_3_-pyrimidine), 3.48–3.50 (m, 4H, morpholine), 3.68–3.71 (m, 4H, morpholine), 7.50 (d, *J* = 8.2 Hz, 2Ar-H), 7.71 (d, *J* = 8.2 Hz, 2Ar-H), 8.17 (s, 1H, CH), 11.63 (s, 1H, NH). ^13^C NMR (75 MHz, DMSO-*d*_6_): *δ*_C_ 23.1, 47.0, 66.2, 128.7, 129.3, 134.1, 134.3, 137.8, 141.1, 142.9, 156.7, 157.0, 164.1. MS (*m*/*z*) = 376 (M^+^). Anal. calcd for C_16_H_17_ClN_6_O_3_ (%): C, 51.00; H, 4.55; N, 22.30. Found: C, 50.98; H, 4.53; N, 22.28.

#### 4-(2-(2-(3-Bromobenzylidene)hydrazineyl)-6-methyl-5-nitropyrimidin-4-yl)morpholine (4e)

Yellow powder; yield = 71%; m. p. 171–172 °C; IR (KBr disc, cm^−1^): *ν* 3580, 3415, 3272, 3051, 2953, 2855, 1572, 1551, 1527, 1486, 1445, 1361, 1323, 1259. ^1^H NMR (300 MHz, DMSO-*d*_6_): *δ*_H_ 2.53 (s, 3H, CH_3_-pyrimidine), 3.67–3.70 (m, 8H, morpholine), 7.36–7.42 (m, 1H, Ar-H), 7.57 (d, *J* = 7.9 Hz, 1H, Ar-H), 7.70 (d, *J* = 7.8 Hz, 1H, Ar-H), 7.85 (s, 1H, Ar-H), 8.15 (s, 1H, CH), 11.66 (s, 1H, NH). ^13^C NMR (75 MHz, DMSO-*d*_6_): *δ*_C_ 23.1, 47.0, 66.2, 122.6, 126.0, 129.4, 131.4, 132.3, 137.7, 142.4, 156.7, 157.0, 164.1. MS (*m*/*z*) = 421 (M^+^), 220 [M^+^ − (bromobenzene, NO_2_)]. Anal. calcd for C_16_H_17_BrN_6_O_3_ (%): C, 45.62; H, 4.07; N, 19.95. Found: C, 45.60; H, 4.05; N, 19.93.

#### 4-(6-Methyl-5-nitro-2-(2-(4-nitrobenzylidene)hydrazineyl)pyrimidin-4-yl)morpholine (4f)

Yellow powder; yield = 65%; m. p. 240–241 °C; IR (KBr disc, cm^−1^): *ν* 3313, 3195, 3064, 2962, 2923, 2865, 1579, 1550, 1508, 1446, 1339, 1253, 1169. ^1^H NMR (300 MHz, DMSO-*d*_6_): *δ*_H_ 2.44 (s, 3H, CH_3_-pyrimidine), 3.49 (t, *J* = 4.7 Hz, 4H, morpholine), 3.68–3.71 (m, 4H, morpholine), 7.91–7.96 (m, 2H, Ar-H), 8.26–8.31 (m, 3H, (2Ar-H, CH)), 11.90 (s, 1H, NH). ^13^C NMR (75 MHz, DMSO-*d*_6_, ppm): *δ*_C_ 23.0, 47.0, 66.2, 124.6, 127.8, 128.9, 132.7, 141.5, 141.7, 147.7, 156.6, 157.1, 163.2, 164.1. MS (*m*/*z*) = 387 (M^+^), 220 [M^+^ − (nitrobenzene, NO_2_)]. Anal. calcd for C_16_H_17_N_7_O_5_ (%): C, 49.61; H, 4.42; N, 25.31. Found: C, 49.59; H, 4.40; N, 25.29.

## Conflicts of interest

The authors declare that they have no conflicts of interest.

## Supplementary Material

RA-016-D6RA01465H-s001

## Data Availability

The datasets used and analyzed in the current study are available in the supplementary information (SI). Supplementary information is available. See DOI: https://doi.org/10.1039/d6ra01465h.
