# Supplementary material for: Metal-free Minisci C–H alkylation of hydrazones using aldehydes: an unexpected route to hydrazone-containing pyrimidine derivatives
Source: RSC Adv. 2026 Apr 29;16(24):22261–8. doi: 10.1039/d6ra01465h (PMC13127404; doi:10.1039/d6ra01465h)

Supplementary Information (SI) for RSC Advances.
This journal is © The Royal Society of Chemistry 2026

Supporting Information

Metal-Free Minisci C–H Alkylation of Hydrazones Using Aldehydes : An Unexpected Route to Hydrazone-Containing Pyrimidine Derivatives

Atefeh Tirehdast[1], Volodymyr Semeniuchenko[2], Ali Shiri[1]*

[1]Department of Chemistry, Faculty of Science, Ferdowsi University of Mashhad, Mashhad, Iran.

[2]Department of Chemistry and Biomolecular Sciences, Faculty of Science, University of Ottawa, Ottawa, Canada.

[*]Corresponding Author E-mail: alishiri@um.ac.ir

Table of contents **pages**

1. ^{1}H, and ^{13}C NMR spectra of compounds (2), (3), (I), (4a-f)…… …………S2-19
2. D_2O-exchangeeable spectrum of compound (4a)……………………………S20

^{1}H NMR spectrum of compound **(2)**

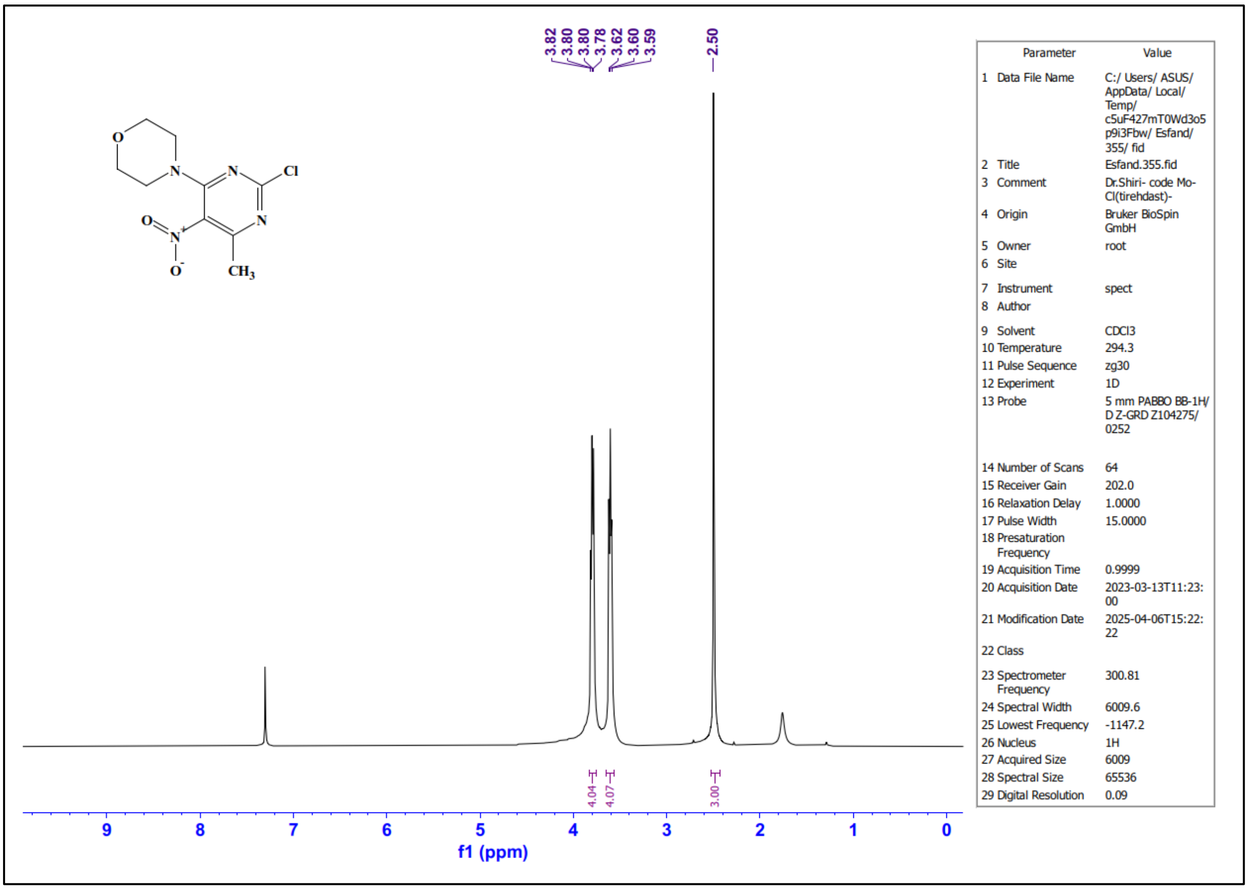

^{13}C NMR spectrum of compound **(2)**

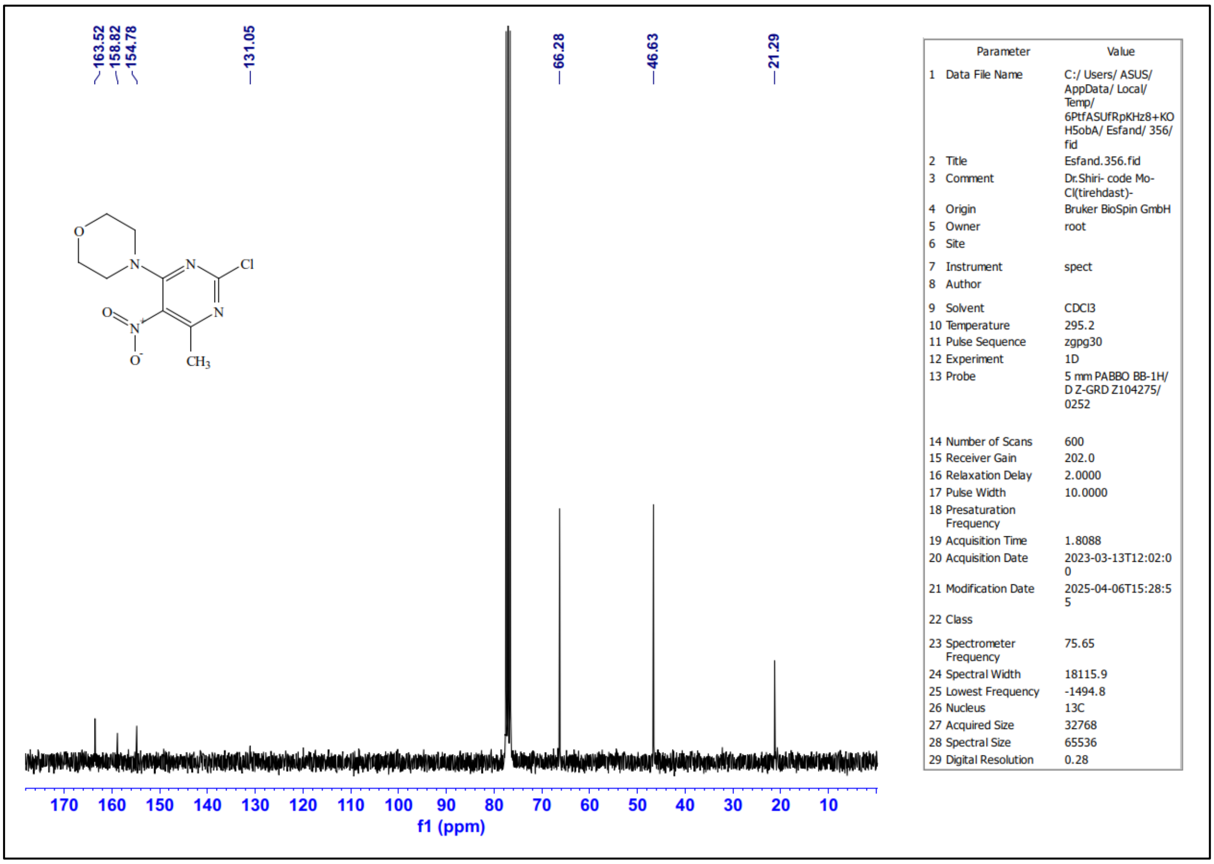

^{1}H NMR spectrum of compound **(3)**

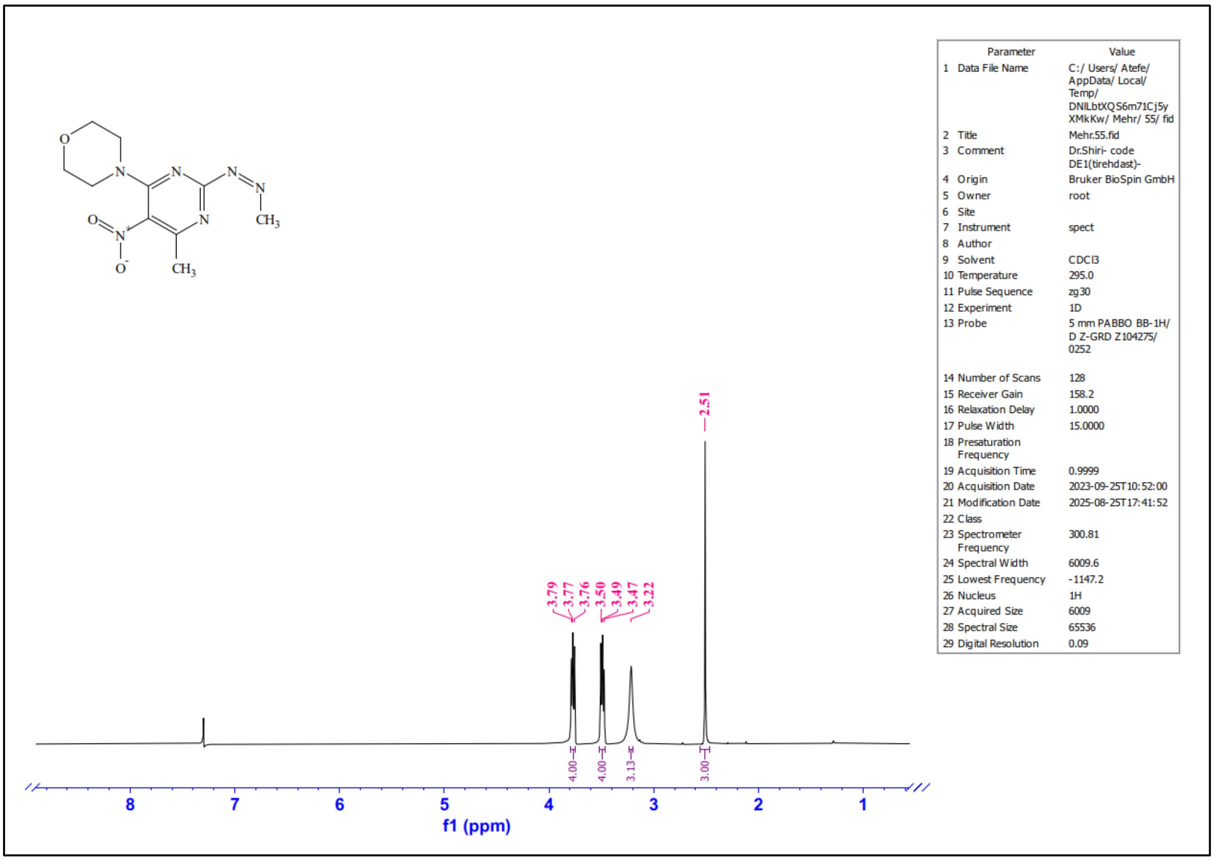

^{13}C NMR spectrum of compound **(3)**

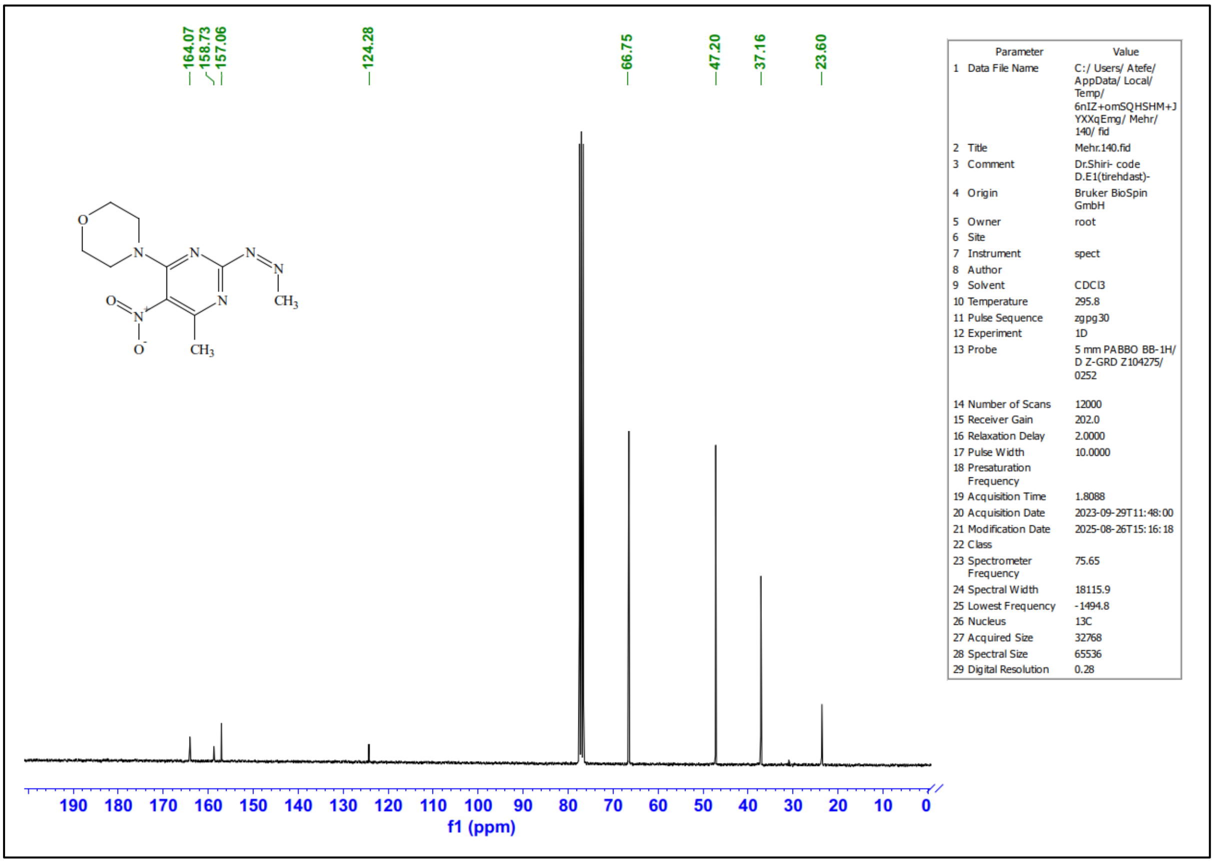

^{1}H NMR spectrum of compound **(I)**

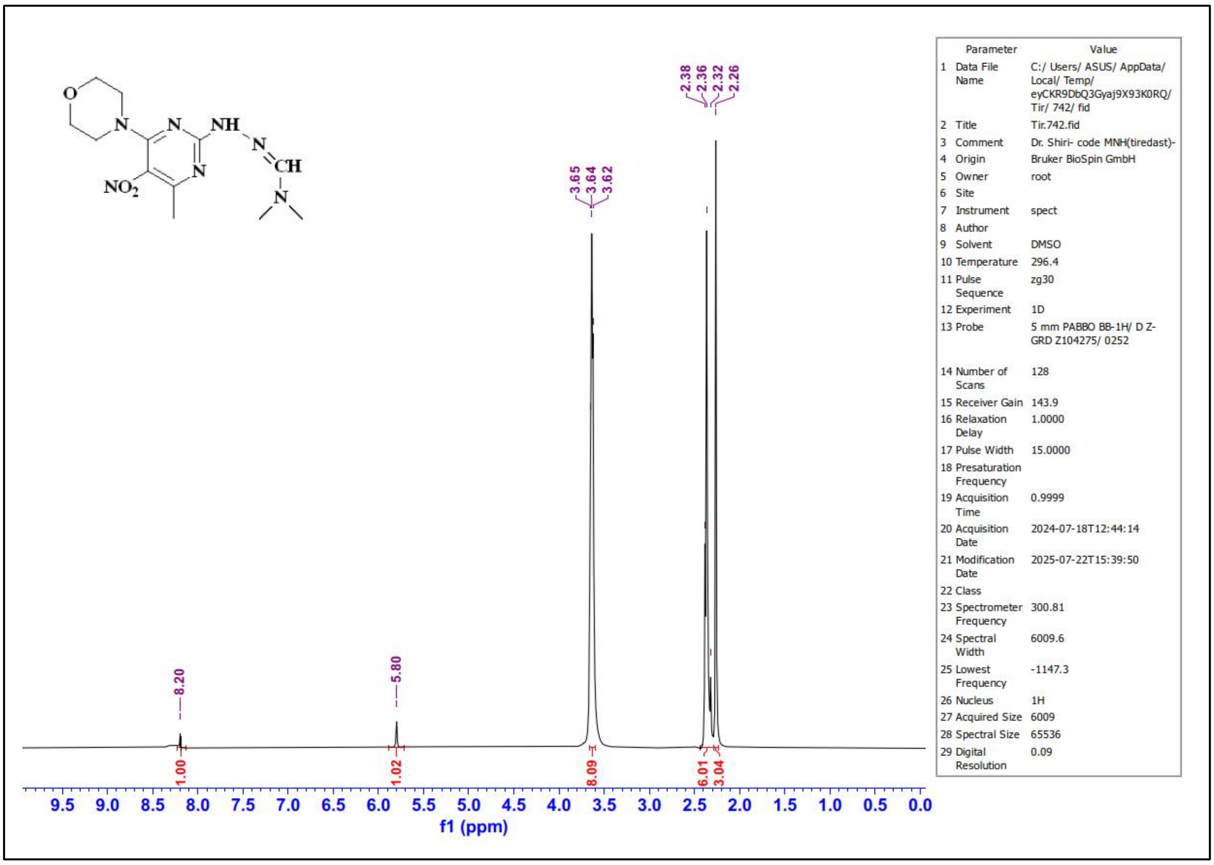

^{13}C NMR spectrum of compound **(I)**

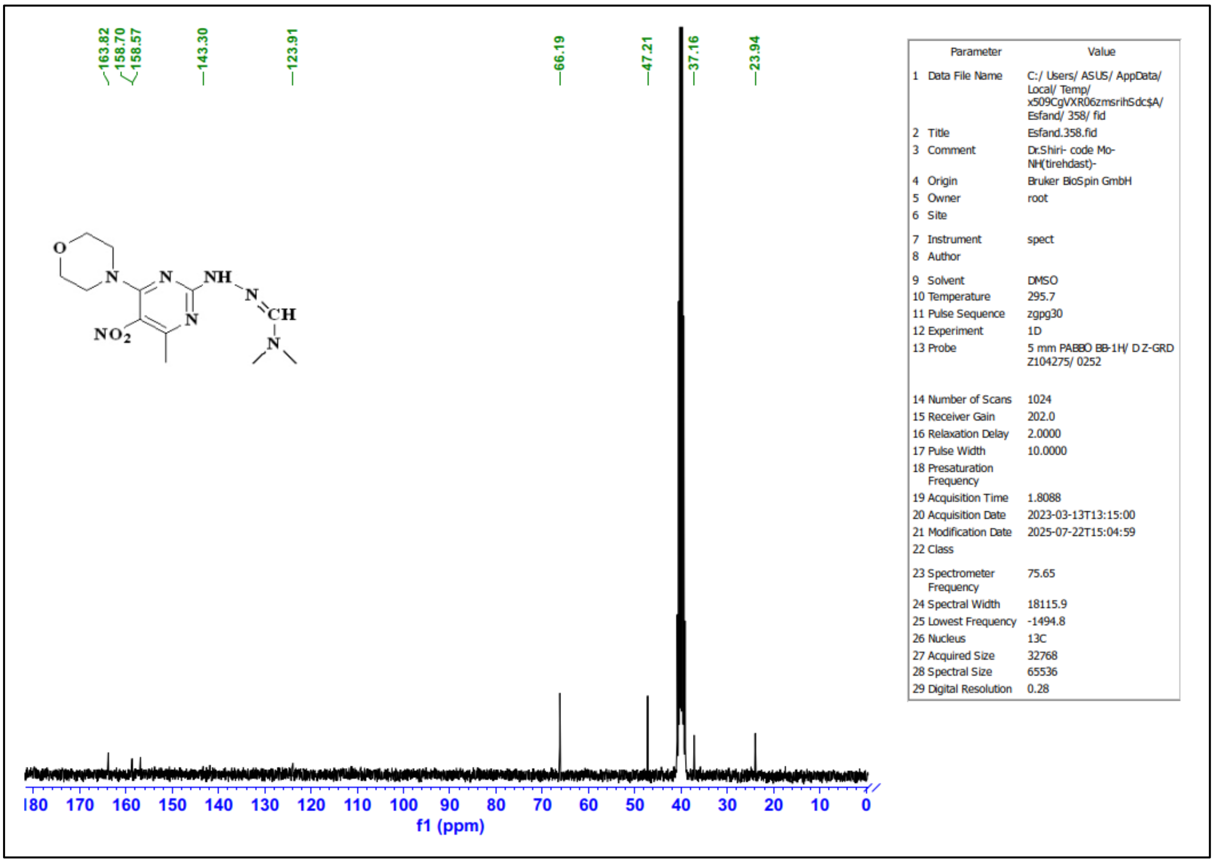

^{1}H NMR spectrum of compound **(4a)**

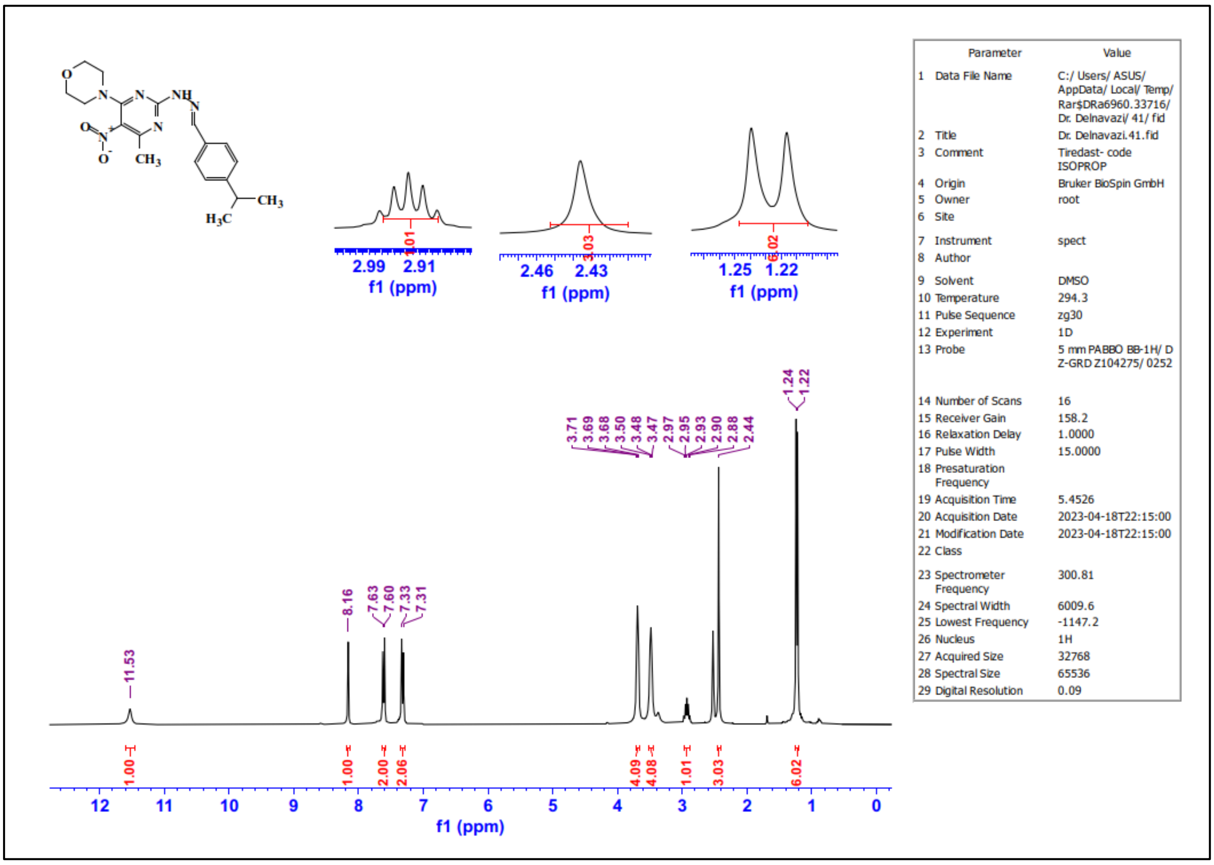

^{13}C NMR spectrum of compound **(4a)**

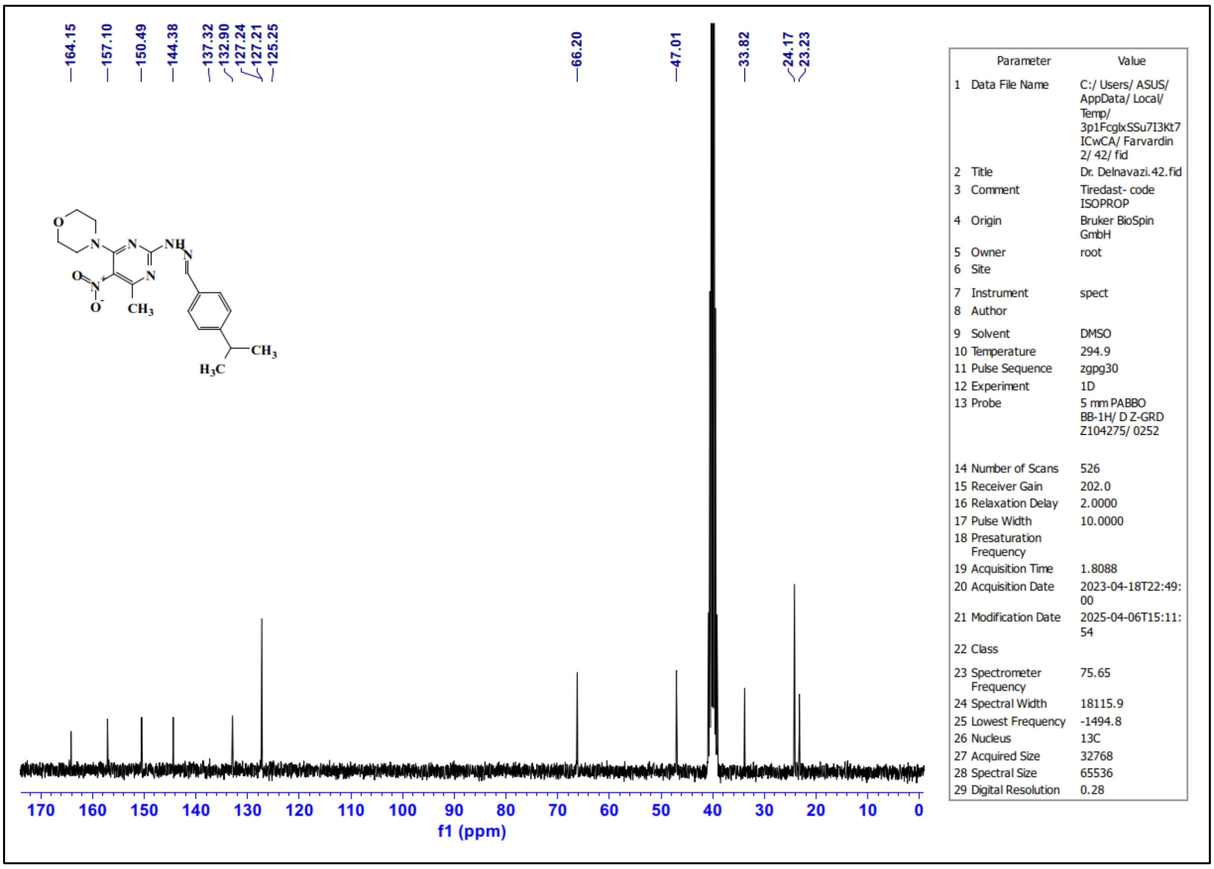

^{1}H NMR spectrum of compound **(4b)**

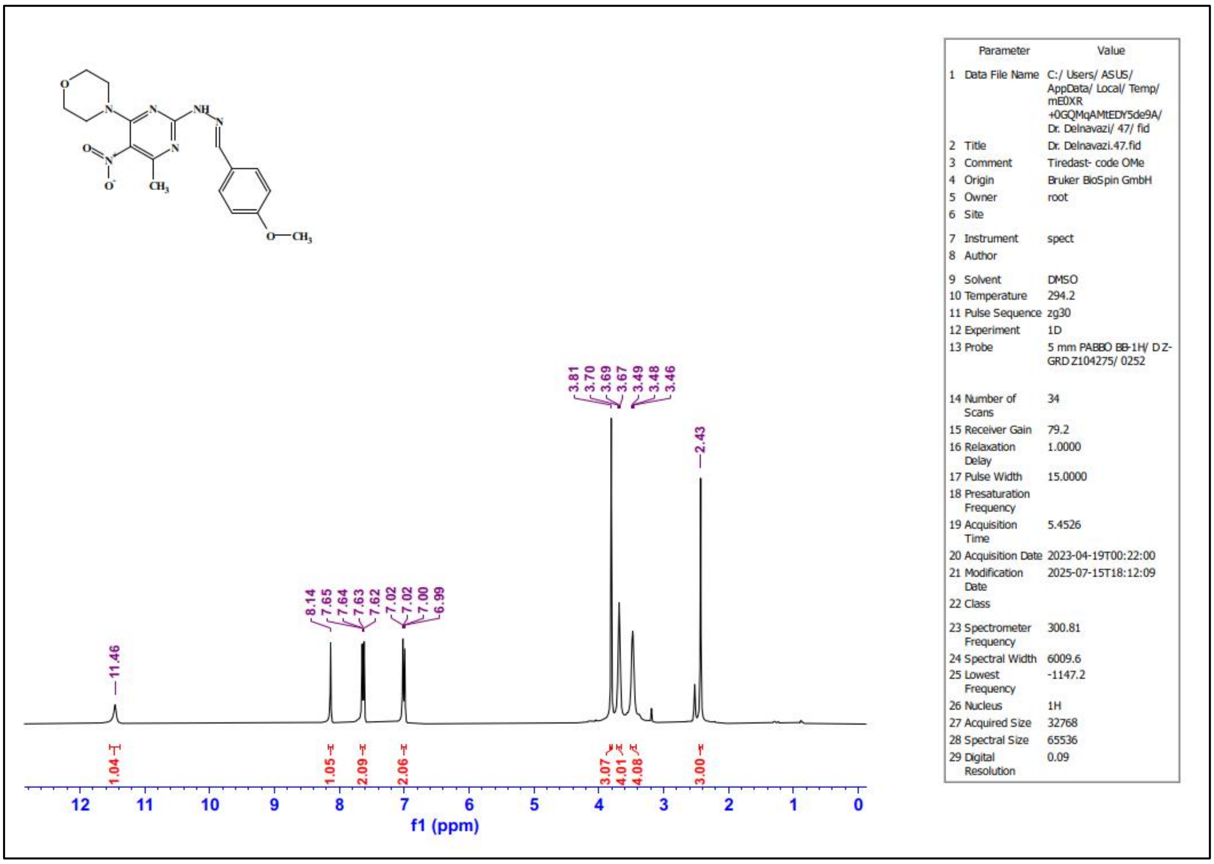

^{13}C NMR spectrum of compound **(4b)**

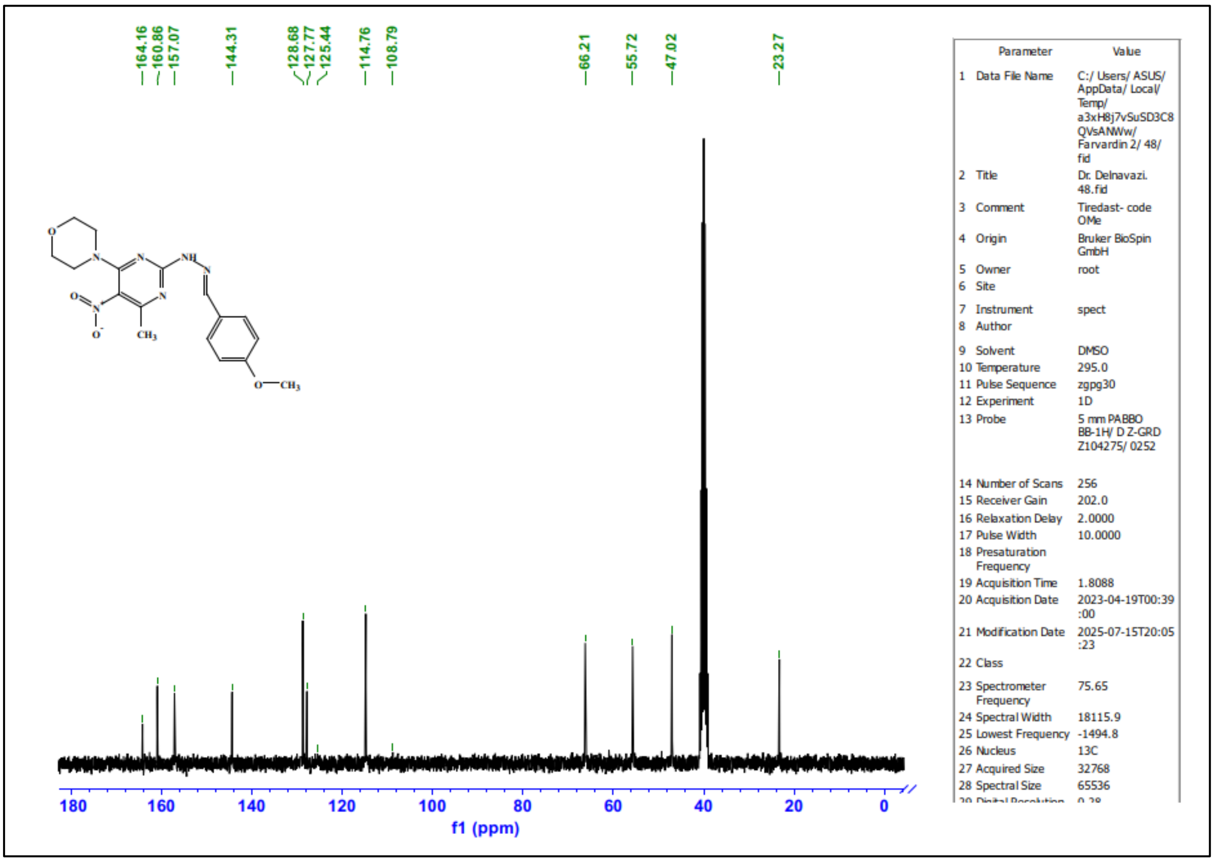

^{1}H NMR spectrum of compound **(4c)**

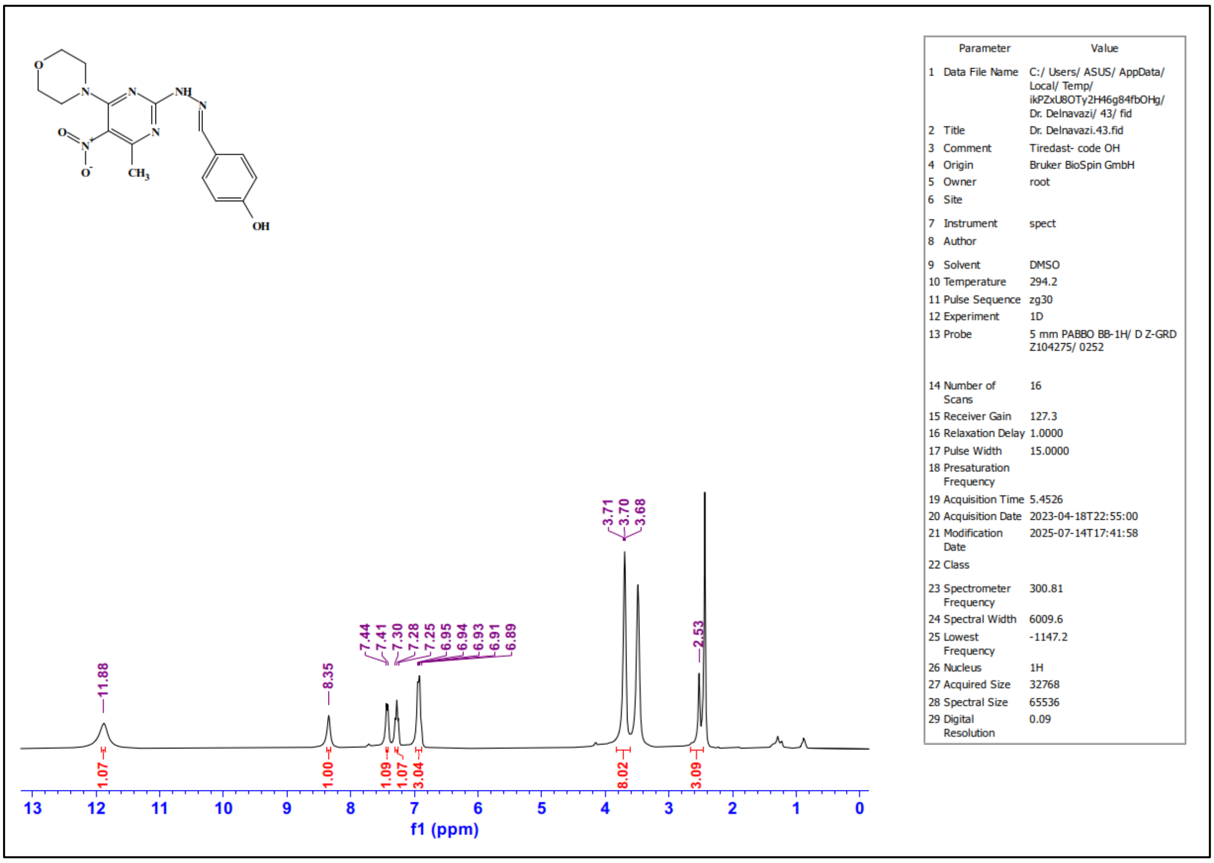

^{13}C NMR spectrum of compound **(4c)**

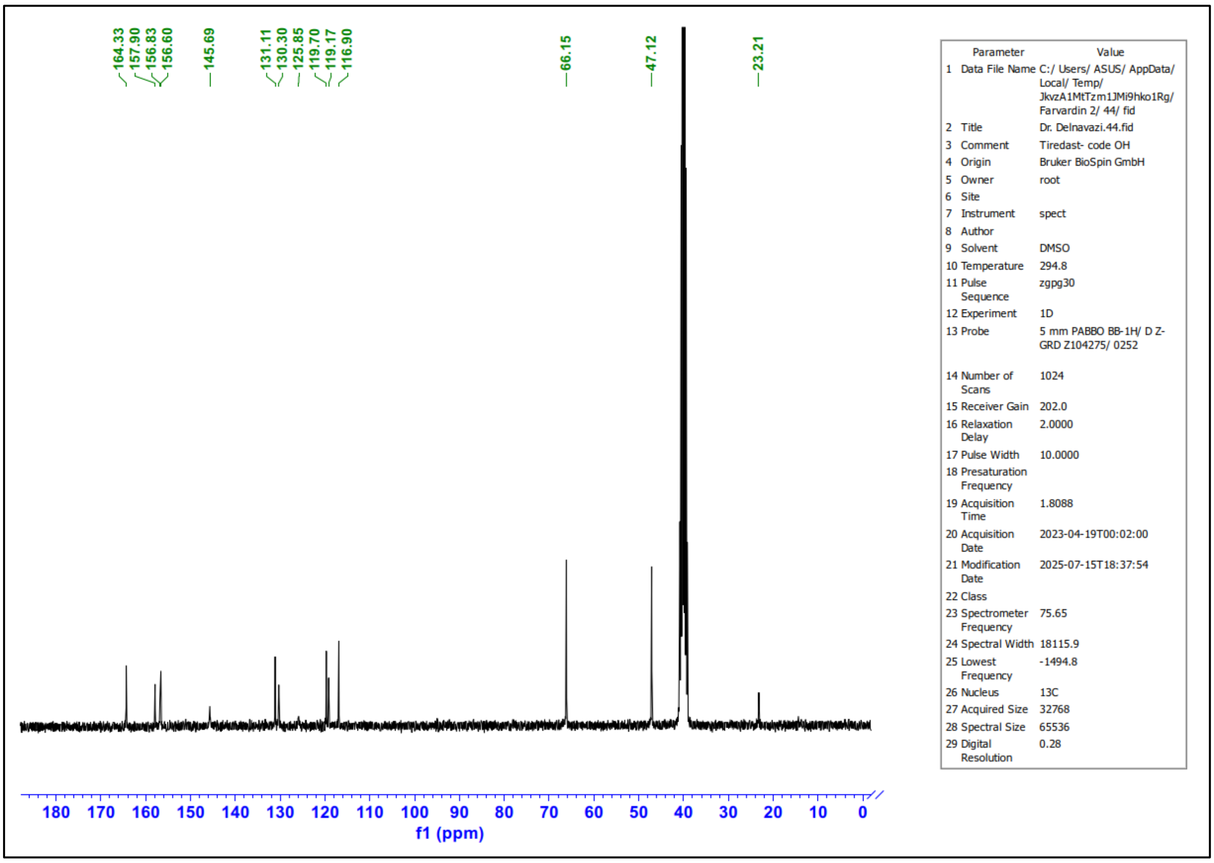

^{1}H NMR spectrum of compound **(4d)**

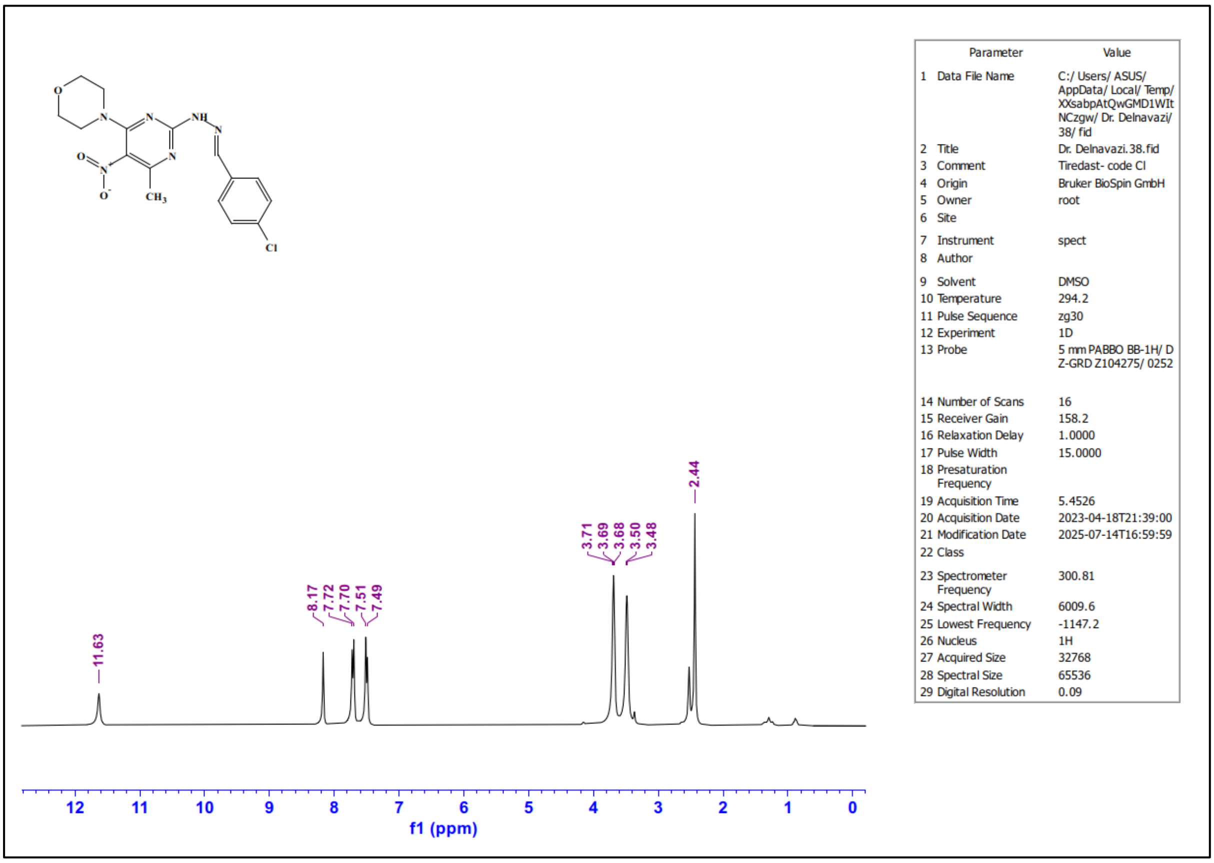

	Parameter	Value
1	Data File Name	C:/ Users/ ASUS/ AppData/ Local/ Temp/ XXsabpAtQwGMD1WIt NCzgw/ Dr. Delnavazi/ 38/ fid
2	Title	Dr. Delnavazi.38.fid
3	Comment	Tiredast- code Cl
4	Origin	Bruker BioSpin GmbH
5	Owner	root
6	Site	
7	Instrument	spect
8	Author	
9	Solvent	DMSO
10	Temperature	294.2
11	Pulse Sequence	zg30
12	Experiment	1D
13	Probe	5 mm PABBO BB-1H/ D Z-GRD Z104275/ 0252
14	Number of Scans	16
15	Receiver Gain	158.2
16	Relaxation Delay	1.0000
17	Pulse Width	15.0000
18	Presaturation Frequency	
19	Acquisition Time	5.4526
20	Acquisition Date	2023-04-18T21:39:00
21	Modification Date	2025-07-14T16:59:59
22	Class	
23	Spectrometer Frequency	300.81
24	Spectral Width	6009.6
25	Lowest Frequency	-1147.2
26	Nucleus	1H
27	Acquired Size	32768
28	Spectral Size	65536
29	Digital Resolution	0.09

^{13}C NMR spectrum of compound **(4d)**

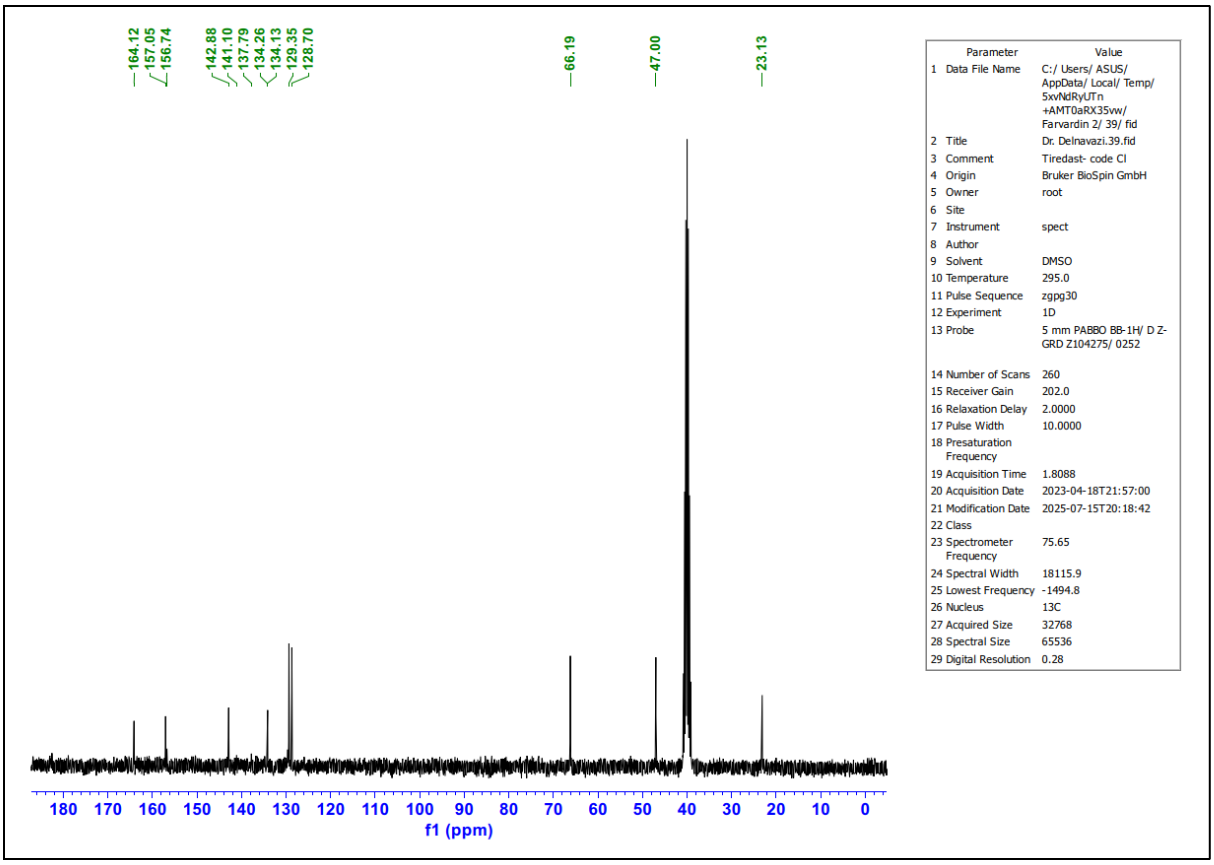

^{1}H NMR spectrum of compound **(4e)**

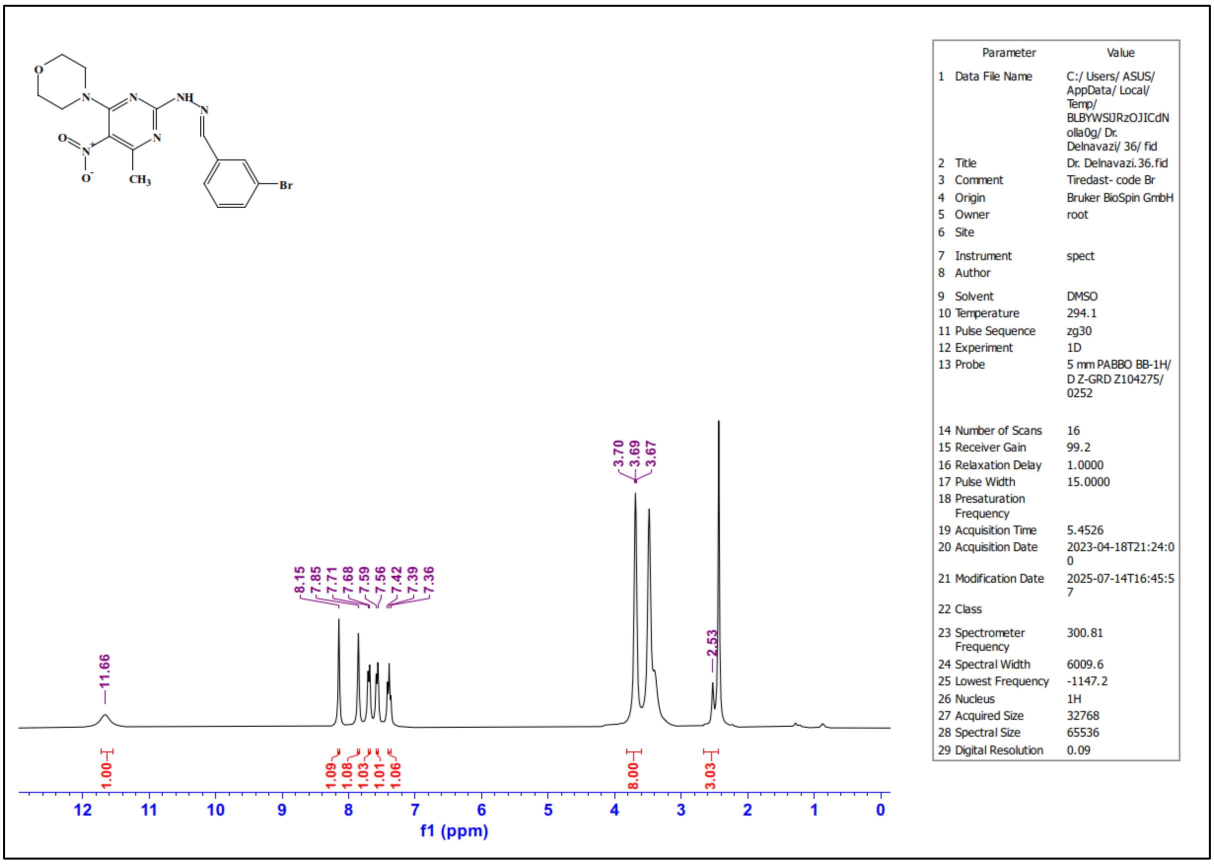

^{13}C NMR spectrum of compound **(4e)**

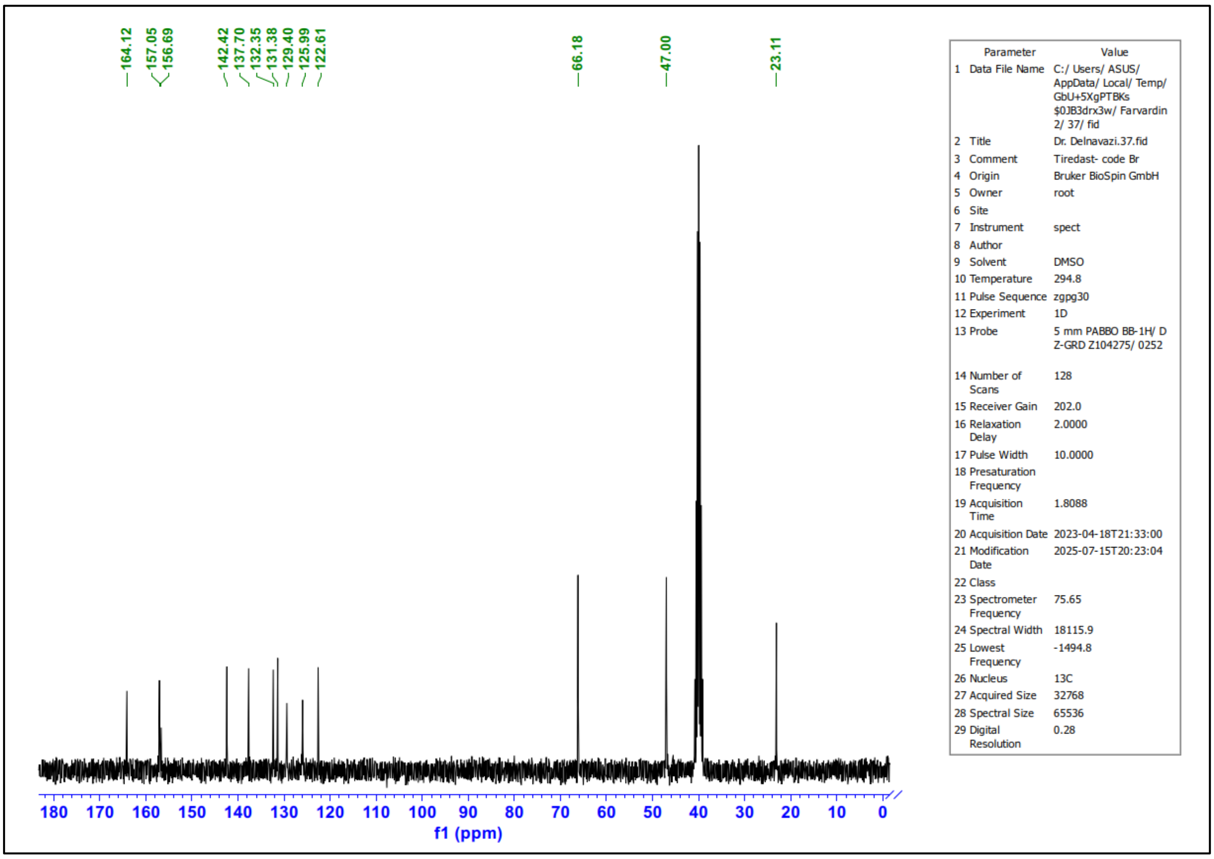

^{1}H NMR spectrum of compound **(4f)**

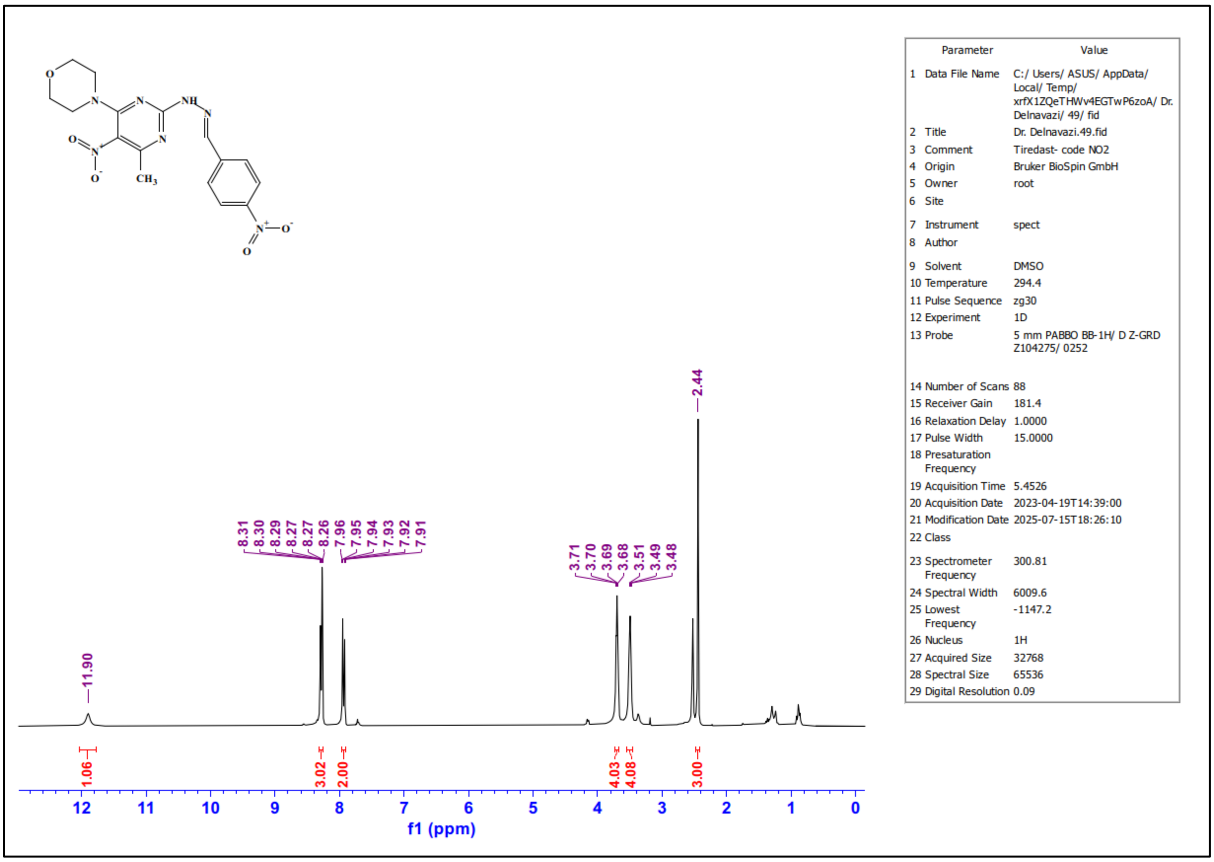

^{13}C NMR spectrum of compound **(4f)**

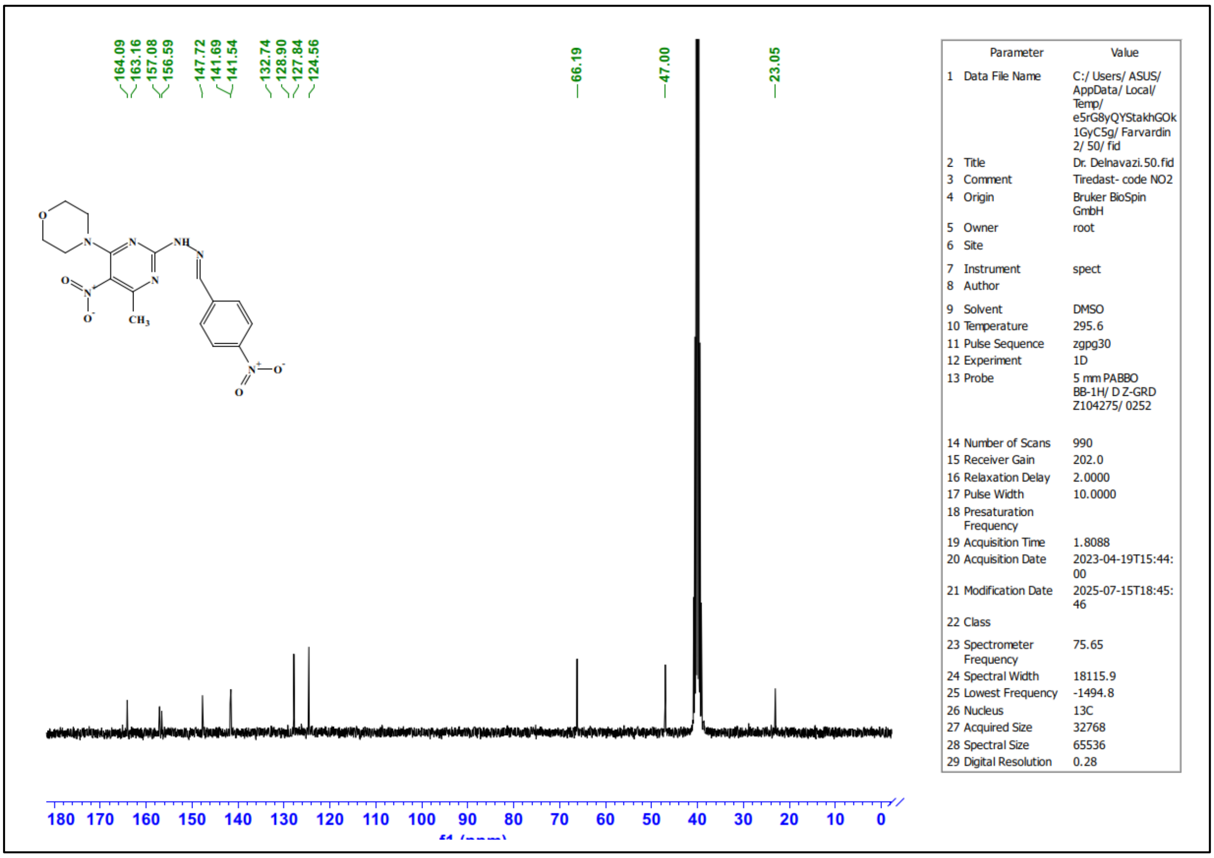

D2O-exchangeable spectrum of compound (**4a**)

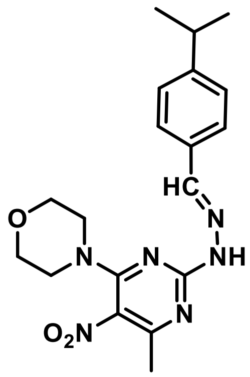

Without D_2O

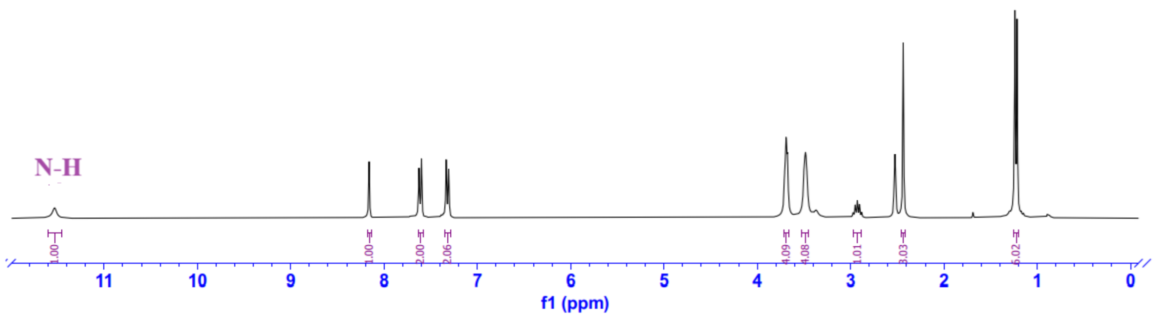

With D_2O

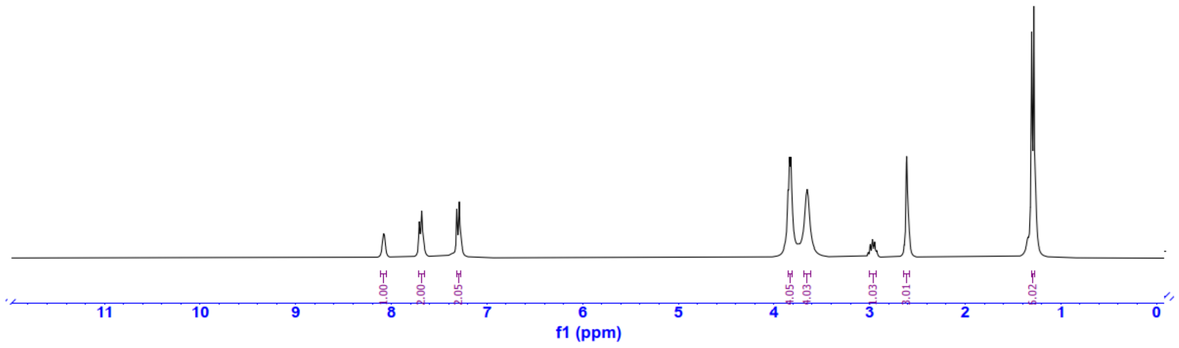

Supplement: RA-016-D6RA01465H-s001 [file RA-016-D6RA01465H-s001.pdf]
